# Looking into Abnormal Co-Expressions of Tau and TDP-43 in the Realm of Mixed Dementia Types: A Double-Punch Scenario

**DOI:** 10.3390/brainsci15070716

**Published:** 2025-07-03

**Authors:** Hossam Youssef, Carina Weissmann, Gokhan Uruk, Rodolfo Gabriel Gatto

**Affiliations:** 1 Department of Neurology, Mayo Clinic, Rochester, MN 55905, USA; youssef.hossam@mayo.edu (H.Y.); uruk-contreras.gokhan@mayo.edu (G.U.); 2 Institute of Physiology, Molecular Biology and Neuroscience, Buenos Aires C1428EGA, Argentina; carinaw@fbmc.fcen.uba.ar

**Keywords:** frontotemporal lobar degeneration, Alzheimer’s disease, tau, TDP-43, neuroimaging, neuropathology, molecular mechanisms, biomarkers

## Abstract

Transactive response DNA-binding protein of 43 kDa (TDP-43) and tau proteins play critical roles in neurodegenerative diseases, particularly frontotemporal lobar degeneration (FTLD) and Alzheimer’s disease (AD). The co-occurrence of TDP-43 and tau pathologies raises questions about their role in disease progression. This review explores the simultaneous presence of tau and TDP-43 co-pathologies, emphasizing their molecular interactions and the resultant neuropathological implications. Additionally, we provide representative examples of their clinical presentations, neuroimaging, and neuropathological findings associated with FTLD-TDP and FTLD-tau, emphasizing the need for a comprehensive understanding of these intertwined pathologies. We analyze various clinical scenarios, including argyrophilic grain disease (AGD), primary age-related tauopathy (PART), and limbic predominant age-related TDP-43 encephalopathy (LATE), to elucidate the complex relationship between these proteinopathies. From the literature, the co-occurrence of tau and TDP-43 is linked to more severe and poorer clinical outcomes compared to isolated pathologies. This review underscores the necessity of considering co-pathologies in the context of FTLD, as they may act as accelerators of cognitive decline. This highlights the importance of integrated approaches in diagnosing and treating neurodegenerative conditions characterized by tau and TDP-43 misfolding. Understanding the interplay between these molecular markers is vital for advancing therapeutic strategies for such disorders.

## 1. Introduction

Dementia syndromes are frequently characterized by multiple neuropathologies, two of which are transactive response DNA-binding protein of 43 kDa (TDP-43) and tau [1,2,3,4]. For instance, up to 57% of Alzheimer’s disease (AD) patients exhibit TDP-43 pathology [2,3]. The reported high frequency of combined pathologies suggests that their interaction might be playing a significant role in clinical presentation and disease pathogenesis [1,5,6,7]. TDP-43 comorbid pathology has been linked to altered clinical features, cognitive decline severity, and faster brain atrophy [2,3,6,8,9,10]. Understanding these interactions and changes is vital for accurate prognostic and management procedures and outcomes.

Research has shown that both TDP-43 and tau do not simply co-exist but interact synergistically, leading to exacerbating neurotoxic effects [9,11,12,13]. Additionally, TDP-43 has been shown to promote tau aggregation [4,13], as well as increase its seeding potentials [6,13]. On the other hand, tau oligomers can induce TDP-43 mislocalization and aggregation [3].

There is increasing evidence demonstrating direct molecular bindings and interactions between TDP-43 and tau proteins [2,3,9,14,15]. Additionally, apolipoprotein E (APOE) ε4 shared genetic pleiotropy, which suggests underlying common mechanisms [6]. In vivo, studies suggest that co-expression or co-introduction of TDP-43 and tau leads to more accelerated accumulation of both pathologies, as well as enhanced neurodegeneration and behavioral deficits [2,6,13,16]. Following the same line, human studies demonstrated that the co-occurrence of TDP-43 and tau pathology is associated with clinical features that are more severe or distinct than those seen in individuals with either pathology alone [2,4,6,11,12,17,18].

For all the above-mentioned findings, we conducted this review to examine TDP-43 and tau co-pathology, shedding light on the molecular aspect, clinical and pathological changes, and discussing the co-pathology effect in different clinical/pathological aspects.

## 2. TDP-43 and Tau: Molecular Perspectives

TDP-43 is a key player in neurodegenerative diseases, particularly in conditions such as frontotemporal lobar degeneration (FTLD)-TDP, Perry syndrome [19,20], and amyotrophic lateral sclerosis (ALS), where its pathological aggregation is a hallmark [21]. Under physiological conditions, TDP-43 is involved in multiple cellular processes, primarily RNA metabolism, including splicing and stability [22,23,24,25]. It interacts with a variety of RNA-binding proteins [26]. It is known to regulate the splicing of specific exons in genes that are critical for neuronal survival, such as the Bcl-2 interacting mediator of cell death (BIM) [22]. Its capacity to shuttle between the nucleus and cytoplasm enables it to participate in various cellular processes, underscoring its dual role in both transcriptional repression and RNA processing [26,27].

TDP-43 abnormal cytoplasmic aggregation is a defining characteristic of FTLD-TDP, Perry Syndrome [19], and ALS [28,29]. It is believed that this aggregation disrupts normal cellular functions, resulting in neurotoxicity. Research indicates that TDP-43 can form toxic oligomers, which are associated with the progression of these diseases [30]. These oligomers are thought to possess prion-like properties, facilitating the dissemination of pathology throughout the brain [30]. Additionally, the phosphorylation of TDP-43 is a significant factor that increases its propensity for aggregation and neurotoxicity, as research has shown that phosphorylated TDP-43 is more prone to forming aggregates [31]. In addition to phosphorylation, various other biochemical modifications, such as N-terminal truncation, C-terminal fragmentation, ubiquitination, acetylation, and sumoylation, may account for the heterogeneity observed in misfolded TDP-43 [32]. This variability supports the concept of TDP-43 strains, potentially elucidating the phenotypic diversity observed in neurodegenerative diseases and hypothesizing the prion-like propagation of TDP-43 [32,33].

Another key protein involved in neurodegenerative diseases is tau, which is essential for neuronal structure and function [34,35]. Tau’s primary function is to stabilize microtubules, which are essential for maintaining neuronal structure and facilitating intracellular transport [36]. However, tau’s role extends beyond microtubule stabilization. Tau has been shown to interact with protein kinase C and Casein kinase substrate in neurons 1 (PACSIN1), which regulates synaptic strength, showing that tau may play a role in synaptic plasticity [37,38]. Interestingly, tau’s localization within the cell significantly influences its functional roles [39]. Traditionally recognized for its association with microtubules in the cytoplasm, recent findings indicate that tau is also present in the nucleus, where it may contribute to gene regulation and chromatin organization [40,41]. This nuclear localization implies that tau’s functions are not confined solely to the cytoskeletal framework; rather, they extend to essential nuclear processes, which could have implications for cellular aging and neurodegeneration [42,43]. Tau proteins in the brain exhibit heterogeneity resulting from alternative splice forms and various post-translational modifications, including phosphorylation [44]. Exon 10 of the microtubule-associated protein tau (*MAPT*) gene undergoes alternative splicing, leading to the production of tau species that contain either three or four conserved approximately 32-amino-acid repeats within the microtubule-binding domain [45,46,47]. These variants are designated as 3-repeat (RD3) and 4-repeat (RD4) tau [48].

Tau accumulation in neurons and glial cells is a well-documented phenomenon [49,50,51], associated with a variety of neurodegenerative diseases and aging processes [52]. Disorders in which tau plays a significant pathological role are classified as “primary tauopathies [52].” Notably, there is a preferential accumulation of 3R tau and 4R tau in many tauopathies, which allows for a biochemical subclassification of these conditions [52]. FTLD-tau encompasses different subtypes of tauopathies, including Pick’s disease, characterized by 3R tau accumulation, as well as corticobasal degeneration (CBD) and progressive supranuclear palsy (PSP), which are associated with the accumulation of 4R tau [53,54]. The detection of these differences has been proposed as diagnostic biomarkers, as the proportion of the 3RT/4RT tau ratio, as well as TDP-43, can be detected in extravesicular vesicles [55].

The co-occurrence of TDP-43 and tau pathologies is increasingly recognized in several neurodegenerative conditions, prompting key questions about their mechanistic interplay and impact on disease progression [6,9,17,56]. Research has indicated that tau tubulin kinases TTBK1 and TTBK2, which are upregulated in tauopathies, have been shown to mediate the pathological phosphorylation of both tau and TDP-43, suggesting they act as molecular links converging on both proteins [57,58]. This dual targeting may enhance co-pathology in vulnerable neurons. Furthermore, the presence of TDP-43 in tau-positive diseases, such as CBD, suggests a potential overlapping pathway [59,60]. This is particularly evident when TDP-43 pathology is localized around tau-positive plaques [60,61]. This supports the notion that tau and TDP-43 may form composite aggregates or facilitate each other’s aggregation.

TDP-43 has been implicated in multiple mechanisms affecting tau pathology. It can exacerbate p-tau accumulation indirectly and enhance its seeding potential [6,62]. Additionally, TDP-43 influences tau expression at the RNA level by promoting tau mRNA instability, which leads to reduced tau protein levels [63]. Moreover, it modulates tau pre-mRNA splicing, favoring the production of the 4R tau isoform [64], which is more prone to aggregation and associated with certain tauopathies such as CBD. In turn, tau pathology may impair nucleocytoplasmic transport, further exacerbating TDP-43 mislocalization and loss of function.

**Figure 1:**
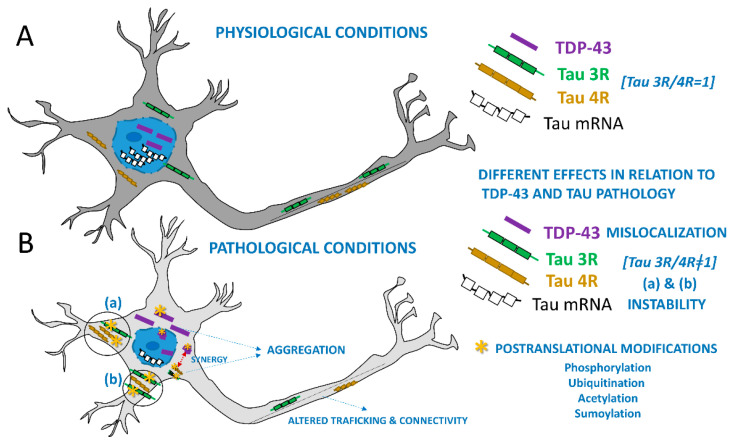
Schematic representation of a neuron under normal and pathological conditions as described in mixed proteinopathies. Key differences between physiological (**A**) and pathological (**B**) conditions include the altered localization of TDP-43, its contribution to tau mRNA instability and the 3R/4R tau ratio, as well as the post-translational modifications observed in tau and TPD-43 in the pathological state including phosphorylations, among others. Note synergistic interactions that drive the misfolding of both proteins.

Different studies have analyzed biochemical factors that contribute to the development of these proteinopathies upstream [4,65]. In addition to the post-translational modifications of tau and TDP-43 found in inclusions [66], in the aging brain, the decrease in chaperone activity is one contributing factor. Biochemical studies suggest that shared upstream insults, such as impaired autophagy, oxidative stress, or reduced chaperone availability, can compromise proteostasis and trigger the initial misfolding of either protein, which then seeds the aggregation of the other [4,56,65,66]. This mutual reinforcement exemplifies the concept of pathologic synergy, where overlapping mechanisms and cellular stress responses drive the co-accumulation of tau and TDP-43 with deleterious downstream effects [56]. Regardless of the mechanism upstream, the accumulation of one misfolded protein triggers the misfolding of different protein species in the same cell, the so-called “pathologic synergy”. Tau and TDP-43 would have the potential for pathologic synergy [6,56], with co-localization of tau and TDP-43 pathologic aggregates in the same cells [67]. This mutual reinforcement exemplifies the concept of pathologic synergy, where overlapping mechanisms and cellular stress responses drive the co-accumulation of tau and TDP-43 with deleterious downstream effects. A schematic representation of this interplay is shown in Figure 1.

## 3. Clinical Presentations and Neuropathological Findings

**Figure 2:**
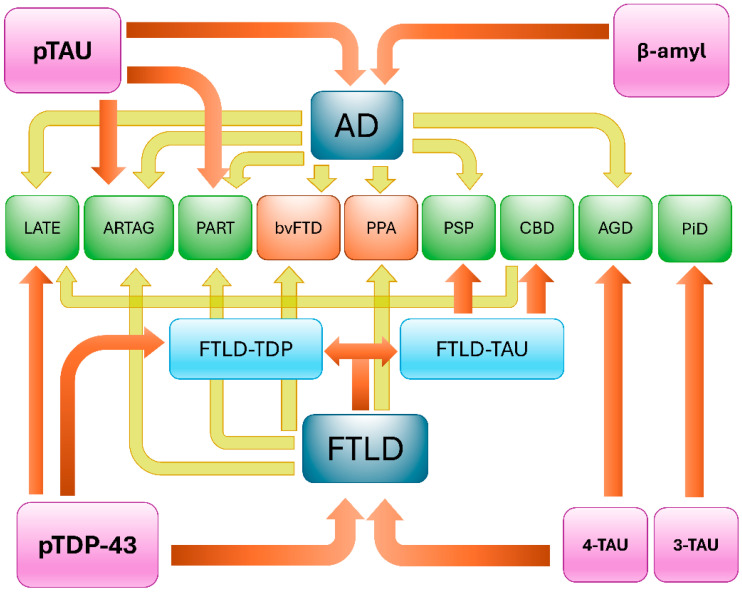
The diagram shows the relationship between the main clinical and pathological attributes in the dementia spectrum and their underlying biochemical features. The relationship between chemical biomarkers (pink boxes) and clinical (orange boxes) or neuropathological (green boxes) phenotypes is pointed out by orange arrows. The possible mixed clinicopathological presentations are marked by the yellow arrows. Note the co-expression of tau and TDP-43 can be observed in the context of Alzheimer’s disease (AD). as well as in Frontotemporal lobar degeneration (FTLD). These mixed types of dementias tend to commonly occur in the elderly where an increased incidence of TDP aggregates in situations like limbic-predominant age-related TDP-43 encephalopathy (LATE) or increasing tau aggregates in aging-related tau astrogliopathy (ARTAG), Primary Age-Related Tauopathy (PART) are present. Abbreviations: pTAU, misfolded phosphorylated tau protein; β-Amyl, misfolded beta-amyloid protein; pTDP-43, misfolded phosphorylated TDP-43 protein; 4-TAU, abnormal tau protein isoform with four microtubule-binding domains; 3-TAU, abnormal tau protein isoform with three microtubule-binding domains; FTLD-TDP, Frontotemporal lobar degeneration with TDP-43 pathology; FTLD-tau, Frontotemporal lobar degeneration with tau pathology; bvFTD, behavioral variant of frontotemporal dementia; PPA, primary progressive aphasia; PSP, progressive supranuclear palsy; CBD, cortico-basal degeneration; AGD, argyrophilic grain disease; PiD, Pick’s disease.

Multiple studies have confirmed that TDP-43 and tau co-pathology are more pronounced in mixed dementia syndromes compared to “pure” dementia syndromes [3,16,68,69]. TDP-43 pathology is found to be higher in Alzheimer’s amnestic dementia syndrome with features of Lewy body dementia [68]. Additionally, the presence of multiple histopathologic diagnoses is common and occurs more frequently in those with dementia compared to those without dementia, and higher numbers of pathologic diagnoses are also associated with greater dementia severity [70]. Hence, the co-occurrence of TDP-43 and tau is often seen in neuropathologic samples of mixed dementia syndromes (Figure 2).

### 3.1. Frontotemporal Lobar Degeneration

From a neuropathological perspective, the co-existence of both proteinopathies leads to complex tissue changes (Table 1). Based on reports from the literature, the co-occurrence of TDP-43 and tau in FTLD is described as rare [59,60]. Nonetheless, when both proteinopathies co-exist, they manifest in several ways. Individuals with mixed FTLD can exhibit both tau-positive and TDP-43-positive inclusions in neurons and glial cells, and the subtypes of FTLD-tau (PiD, CBD, PSP) and FTLD-TDP (type A, B, C) can vary in these mixed cases [60]. A mixed pathology frequency of 50% was reported in cases with FTLD-TDP-43 type A [1]. Additionally, these mixed cases tend to show a higher burden of AD pathology, such as amyloid-β (Aβ) and neurofibrillary tangles (NFTs), although this is not always the case [60]. On the other hand, some studies have found very limited interaction between TDP-43 and tau [61]. It is important to note that while the co-pathology of TDP-43 and tau can occur in FTLD, it might be causatively independent in many examples and could reflect overlapping changes associated with advanced age or genetic risk factors such as the APOE ε4 allele [61].

**Table 1:** Prior relevant literature describing the co-occurrence of TDP-43 and tau proteinopathies in FTLD.

Study	Institution	Study Criteria	Total Cases	Tau Cases	TDP Cases	FUS Cases	Other Cases	Neuropathological and Clinical Findings
Hodges et al., 2004 [71]	Sydney, Australia, and Cambridge, UK	Pathologically confirmed FTLD or CBD from dementia clinics. No PSP	61	31	30	0	0	One out of nine CBD had ubiquitin-positive. CBD pathology was present in seven of nine cases (78%), with one each having FTLD-MND and DLDH.
Kertez et al., 2005 [72]	London, Ontario, Canada	Clinical diagnosis of FTD, CBS, or PSP that went to autopsy	60	21	24	0	15	A case exhibited features of both CBD and MNDI, suggesting transitional patterns between tau-positive and tau-negative types. Clinical CBS with non-CBD pathologies highlights this overlap, making it difficult to predict pathological variety from clinical phenotype. This indicates that initially distinct syndromes may converge in a patient, representing different histological varieties.
Forman et al., 2006 [73]	Philadelphia, PA, USA	Clinical diagnosis of dementia, as well as FTLD, CBD, or PSP pathologic diagnosis	90	53	37	0	0	Progressive aphasia is linked to tau-negative pathologies like FTLD-U, while progressive nonfluent aphasia is associated with PiD or CBD. No specific clinical association between language disorder and pathological subgroups was found. Tauopathy patients often show extrapyramidal features indicative of CBD and PSP, aligning with smaller studies that connect rigidity to CBD and pyramidal features of clinical MND to FTLD-U.
Snowden et al., 2007 [74]	Manchester, UK	Pathologic diagnosis of FTLD or CBD from a cerebral function unit	65	25	40	0	0	One case of tauopathy exhibited CBD-related immunohistochemical changes, despite presenting typically as bvFTD without Parkinsonism or asymmetric apraxia. This highlights that predictable patterns do not guarantee a direct link between clinical and pathological phenotypes.
Robinson et al., 2014 [61]	Manchester, UK	Pathological diagnosis of FTLD-tau, FTLD-TDP, and MND	78	33	45	0	23	In 33 FTLD-tau cases, IHC for non-pTDP-43 showed normal nuclear staining in all but two cases with pathological TDP-43 (NCI, DN, NII). One case had a MAPT+13 mutation with TDP-43 in a few surviving cells and extensive involvement of the entorhinal cortex and fusiform gyrus, alongside Aβ deposition. The CBD case had few NCI and DN in similar regions. Tau immunostaining revealed AT8-positive neurons in 60% of FTLD-TDP patients, 30% of MND patients, and 81% of controls. TDP-43 pathology resembled FTLD-TDP type A but was limited to medial temporal lobe structures, suggesting a secondary pathology akin to changes in elderly AD patients.
Kim et al., 2018 [60]	University of California, LA, USA Pusan national university, Busan, Republic of Korea	Neuropathologic diagnosis of FTLD-TDP and FTLD-tau	9	4	5	0	0	Primary FTLD-TDP presents with unclassifiable FTLD-tau inclusions and conditions like PSP. Mixed cases of FTLD-TDP and FTLD-tau exhibited widespread tau pathologies distinct from Alzheimer’s disease. Among five mixed cases, three showed unclassifiable tauopathy, while two with FTLD-TDP type C were associated with FTLD-tau and PSP. Additionally, TDP-43 pathology was found in four FTLD-tau, CBD cases, with no significant clinical differences between mixed and pure FTLD-tau groups. FTLD-CBD emerged as the predominant form with TDP-43 pathology, primarily affecting regions beyond limbic areas, correlating with clinical symptoms such as abnormal behaviors and nonfluent aphasia.
Pennington et al., 2020 [5]	Bristol & Edinburgh	Neuropathologic diagnosis of FTLD-TDP and FTLD-tau	515	139	359	7	10	In nine cases, primary tauopathy and secondary TDP-43 deposition were noted; four cases had a primary FTLD-TDP diagnosis with additional tauopathy. Mixed neuropathology occurred more frequently in FTLD-TDP (49%) than in FTLD-tau (24%).
Koga et al., 2021 [59]	Jacksonville, Florida, USA	Pathologically confirmed cases of FTLD-TDP with and without MND	201	0	146	0	55	In the FTLD-TDP cases (*n* = 146), PART was found in 34% (50), ARTAG in 44% (64), AGD in 23% (33), and CBD in 1% (2).

Footnote: The table summarizes prior literature on the co-occurrence of TDP-43 and tau proteinopathy in FTLD-spectrum disorders. Acronyms: AD—Alzheimer’s disease; AGD—Argyrophilic grain disease; CBD—Corticobasal degeneration; DLDH—Dementia lacking distinctive histology; FTLD—Frontotemporal lobar degeneration; FTLD-MND—Frontotemporal lobar degeneration with motor neuron disease; FTLD-Tau—Frontotemporal lobar degeneration with tau pathology; FTLD-TDP—Frontotemporal lobar degeneration with TDP-43 pathology; FUS—Fused in sarcoma; MAPT—Microtubule-associated protein tau; MND—Motor neuron disease; NCI—Neuronal cytoplasmic inclusion; PART—Primary age-related tauopathy; PSP—Progressive supranuclear palsy; Tau—Tau protein pathology; TDP-43—TAR DNA-binding protein 43.

In light of the conventional understanding, FTLD typically presents with either TDP-43, FUS, or tau aggregates, with minimal co-occurrence. Therefore, a low prevalence of concurrent TDP-43 and tau co-pathology is anticipated. However, we assert that the employed pathological criteria and study methodology significantly influence the detection of TDP-43 and tau co-occurrence in FTLD. One critical factor is the reliance on traditional approaches that focus predominantly on mature NFT detection markers, which may overlook earlier stages of tau pathology that could coexist with TDP-43. Studies that rely solely on traditional tangle markers may systematically underestimate tau pathology in mixed cases. Reliance on antibodies that identify early tau pathology, such as PHF-1, is crucial. We believe that the PHF-1 tau preferential accumulation in synaptic mitochondria and correlation with oxidative damage and mitochondrial dysfunction indicates that PHF-1 tau may contribute to synaptic dysfunction before the formation of mature tangles becomes apparent [75]. Moreover, PHF-1 promotes specific three-layered β-strand folds that are different from other tau conformations [76], suggesting a distinct pathological entity with unique properties that could facilitate co-occurrence detection with other proteinopathies, including TDP-43. Additionally, sampling strategies and regional analysis patterns also play a role. This is evident when TDP-43 in mixed cases often shows restricted distribution patterns with limitation to specific areas such as the dentate gyrus of the hippocampus and entorhinal cortex in approximately 75% of cases, while 25% demonstrate more widespread inclusions in frontal and temporal cortices [77]. This heterogeneity of distribution patterns means that limited sampling protocols may miss concurrent pathologies that are present yet localized to unexamined regions.

**Figure 3:**
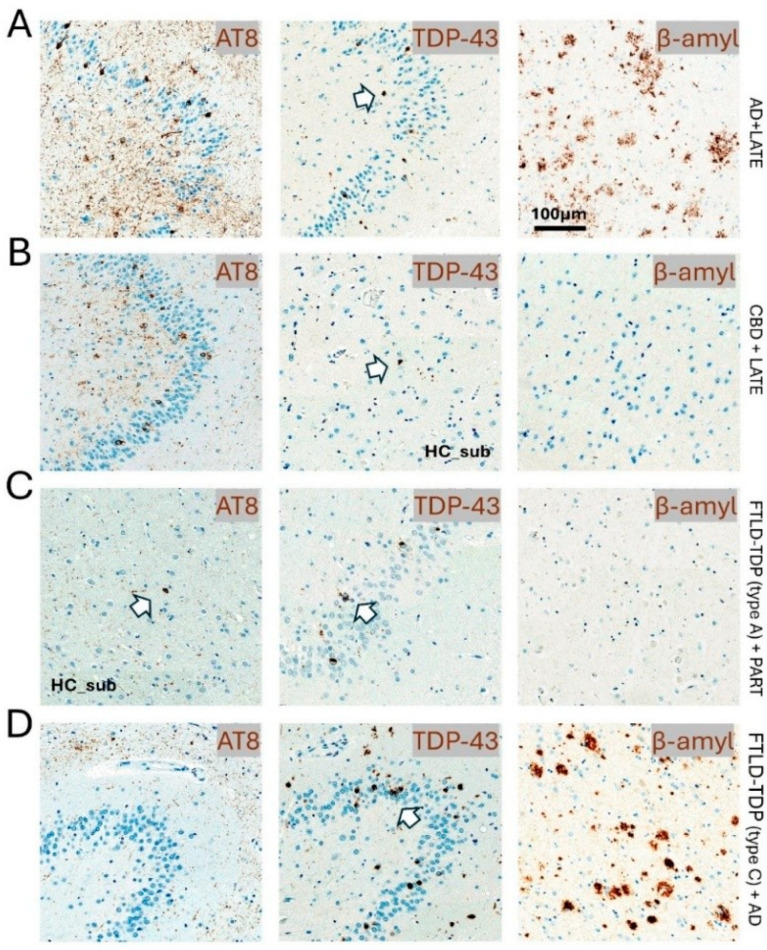
Representative neuropathological examples of mixed tau and TDP proteinopathies. (**A**) Immunohistochemical staining of the hippocampal dental gyrus staining with phosphorylated tau (AT8) and phosphorylated TDP (TDP-43), which are shown in white arrows. Additional beta-amyloid (β-amyl) staining from the inferior temporal cortex is presented. Other co-pathologies include CBD + AD (**B**), FTLD-TDP-43 (type a) +PART (**C**), and FTLD-TDP-43(type c) +AD (**D**). Hematoxylin is used as a nuclear counterstaining (blue). Scale bar = 100 microns.

Although the co-pathology of both TDP-43 and tau in FTLD can be associated with some differences in clinical changes, a completely distinct clinical syndrome cannot be solely attributed to their mixed co-pathology [78]. Studies of mixed pathology cases revealed more widespread brain atrophies compared to pure pathological cases [60], as illustrated in a subset of cases from Figure 3. Moreover, research suggests a potential link between genetic risk factors, AD-related changes, and the co-occurrence of TDP-43 and tau in FTLD, which could indirectly influence clinical presentation, especially if AD pathology contributes to cognitive decline [60]. Surprisingly, a recent study revealed that individuals with FTLD-TDP-43 type A with combined pathology—encompassing hippocampal sclerosis, Alzheimer’s disease-neuropathologic change (ADNC) (sometimes with co-occurring FTLD-tau/PSP), vascular disease, motor neuron disease, and mammillary body atrophy, ranked from highest to lowest prevalence—showed significantly longer lifespans and longer disease durations than those with only FTLD-TDP-43 type A [1]. This finding presents a challenge to the prevailing assumption that co-pathologies consistently lead to worse outcomes. Several interrelated factors may elucidate this unexpected observation. Patients who live longer inherently have more time to develop additional pathologies, suggesting that the most compelling explanation lies in age-dependent pathology accumulation and the inherent survival bias. This creates an apparent association between combined pathology and longevity that may be correlational rather than causal. Notably, the study revealed that individuals with FTLD-TDP type A and combined pathology (mean age at death 73.36) are significantly older than those with isolated pathology (mean age at death 65.67), with no significant difference at the age of onset [1]. Another critical perspective can be observed in the relationship between genetics and combined pathology. Cases exhibiting FTLD-TDP type A with known genetic mutations (e.g., GRN, C9orf72, TMEM106B) generally do not present with combined pathology and demonstrate a significantly younger age of onset and mortality compared to sporadic cases [1]. This observation indicates that genetically driven FTLD-TDP may follow a more aggressive and rapidly progressive course, potentially limiting the development of age-related co-pathologies. In contrast, sporadic cases characterized by slower progression may represent a fundamentally different disease process, exhibiting a greater propensity for pathological heterogeneity and extended survival. On the contrary, this might be explained by the notion of pathological competition and negative interactions, the ceiling effect of pathological burden, or the neuroprotective co-pathology hypothesis [79,80]. In consideration of this, it is important to acknowledge that mixed neuropathology may significantly impact the clinical phenotype of FTLD, resulting in both positive and negative outcomes, thereby representing one of the most challenging aspects of neurodegeneration research, with outcomes that can be counterintuitive and difficult to predict, further complicating accurate antemortem diagnosis [5]. The presence of multiple pathologies can result in a more intricate or overlapping array of symptoms that do not align precisely with the established FTLD subtypes, thus hindering the clinical identification of the specific underlying proteinopathies [5].

The reliance on clinical diagnostic criteria for FTLD classification introduces challenges in accurately identifying mixed pathology cases, as the clinical presentation may not predict the complexity of underlying pathology. For instance, studies examining the clinicopathological correlation in behavioral variants of FTD have reported common co-existing pathology, with 54% of patients with primary FTLD pathology demonstrating some degree of ADNC [81]. The complexity of this issue is further compounded by the observation that various pathological subtypes may exhibit remarkably similar clinical and neuroimaging profiles. This phenomenon is particularly evident in the analysis of specific FTLD histopathological subtypes, where significant overlap in core clinical criteria, symptoms, and additional cognitive and behavioral characteristics wasobserved, alongside convergent patterns of grey matter atrophy across multiple pathological subtypes [81]. This leads us to the conclusion that relying predominantly on clinical diagnosis is impractical, as it tends to underestimate the actual prevalence of mixed pathologies.

### 3.2. Argyrophilic Grain Disease and TDP-43

TDP-43 has been found to co-occur with AGD in the form of small, dense, rounded neuronal cytoplasmic inclusions (NCIs) with few short dystrophic neurites (DNs), primarily observed in limbic regions, superior temporal gyrus, striatum, and midbrain, with the absence of neuronal intranuclear inclusions [82]. TDP-43 was also observed to co-localize with tau within argyrophilic grains and neuronal cell bodies, suggesting a specific pattern of TDP-43 association in the presence of AGD tauopathy [60]. While some CBD cases have co-occurring AGD, the distribution of TDP-43 in these cases was widespread, extending beyond limbic regions such as the middle frontal gyrus and inferior temporal gyrus [5,60]. Another study indicated that cases exhibiting TDP-43 demonstrate greater overall argyrophilic grain density [83]. Furthermore, it was found that cases with elevated argyrophilic grain scores are more likely to present positive pathologic TDP-43 staining compared to those with lower scores [83].

TDP-43 depositions are correlated with the presence of AGD in cognitively normal elderly individuals [5,83]. In addition, amnestic symptoms have been explained by the presence of diffuse AGD and medial temporal lobe TDP-43 proteinopathies [82]. While diffuse AGD typically presents with significant cognitive decline, including executive dysfunction, in addition to amnestic features, the presence of co-localizing TDP-43 as additive pathology could potentially exacerbate or modify the pattern of cognitive decline [82,84]. However, a definitive confirmation is not possible due to the absence of a distinctive clinical syndrome and the lack of a characteristic phenotype for AGD itself, which further complicates the challenge of attributing symptoms to TDP-43.

The frequency of TDP-43 pathology in AGD is dramatically discrepant between studies. In one comprehensive study, 33% of AGD cases exhibited TDP-43 in at least one brain region examined, compared to 24% of age-matched controls, though it was not statistically significant [85]. This sharply contrasts with an earlier study that reported a substantially higher TDP-43 prevalence of 60% in AGD cases [86]. This discrepancy can be attributed to age-related factors, as age emerges as the most significant risk factor for TDP-43 pathology in AGD cases [85]. In discussing the methodological and technical aspects, it is important to note that the selection of antibodies, the number of antibodies utilized, and the detection protocols implemented contribute significantly to the variations observed in the detection of TDP-43 in AGD. Furthermore, it is noteworthy that cases demonstrating TDP-43 were categorized into more advanced stages of AGD in comparison to those that did not exhibit TDP-43 [86]. Also, the progression of AGD pathology may contribute to the accumulation of TDP-43 [86]. Therefore, pathological staging and severity may significantly influence the co-occurrence of TDP-43 in AGD cases.

### 3.3. Corticobasal Degeneration and TDP-43

Many studies have reported TDP-43 pathology and immunoreactivity in CBD cases [5,59,60,77,83]. The most comprehensive systematic study identified TDP-43 pathology in 45% of 187 autopsy-confirmed CBD cases, with the midbrain tegmentum being the most frequently affected region (80% of TDP-43-positive cases), followed by the subthalamic nucleus (69%) [87]. However, the frequency of TDP-43 in CBD varies across studies, ranging from 9% to 90% in different cohorts [74,87,88]. This variability reflects several critical factors that dramatically influence detection rates. The hierarchical clustering analysis of the largest cohort revealed that TDP-43-positive CBD cases could be further subdivided into TDP-limited (52%) and TDP-severe (48%) categories, suggesting that the severity distribution itself affects overall detection frequencies [87]. Additionally, in cohorts primarily focused on FTLD-tau, a subset of CBD cases shows TDP-43 [5,60,77]. Conversely, in primary FTLD-TDP cohorts, coexisting CBD is less common, although it has been documented [59,89]. This frequency disparity arises from cohort selection biases (tau vs. TDP-43 focus), anatomic screening preferences (cortical vs. brainstem), pathogenic interactions (tau predisposing to TDP-43), and genetic compartmentalization. These factors underscore the need for unified neuropathological protocols that assess both pathologies across all regions, particularly in atypical and overlapping syndromes.

The typical lesions of CBD (astrocytic plaques, tau threads, coiled bodies) are present alongside TDP-43 inclusions. These TDP-43 inclusions are predominantly NCIs but can also include DNs and neuronal intranuclear inclusions (NIIs) [60,88,90]. Additionally, TDP-43 pathology in CBD can manifest as FTLD-TDP type A or type B and sometimes as unclassifiable TDP-43 pathology [60,90]. Although TDP-43 distribution in FTLD-CBD cases is widespread, extending beyond limbic areas to regions such as the middle frontal gyrus, inferior frontal gyrus, and inferior temporal gyrus [60], it is not as consistently reported as a primary characteristic of TDP-43 distribution in AGD [83,86].

While the clinical effect of TDP-43 in CBD is complex and still under investigation, there is a tendency for CBD cases with severe TDP-43 pathology to be misdiagnosed with PSP compared to those with CBD with minimal or no TDP-43 [87]. The misdiagnosis is often linked to the presence of downward gaze palsy, a characteristic feature of PSP, which was found to be more frequent in TDP-severe CBD cases and associated with severe TDP-43 pathology in the midbrain tectum [87], which suggests a distinct clinicopathologic subtype of CBD presentation. However, the overall clinical phenotype of CBD cases with TDP-43 was similar to that seen in pure FTLD [87]. Although TDP-43 pathology was found in the spinal cord motor neurons of a significant proportion of CBD cases, there was no significant association between this spinal cord TDP-43 pathology and clinical duration or age at death in PSP/CBD [12].

### 3.4. TDP-43 in Alzheimer’s Disease and Primary Age-Related Tauopathy

In the studies reviewed, TDP-43 presence in PART cases ranged from 29% to 100% [8,10,14,91,92], while AD was observed to be between 19% and 75% [2,3,7,8,14,93,94,95]. From a neuropathologic standpoint, TDP-43 appears to exacerbate tau pathology, leading to increased tau burden and seeding potential in PART and AD [2,3,6,14,96]. This suggests a common pathway where TDP-43 influences tau pathology in both PART (where amyloid is minimal) and AD (where amyloid is significant). Interestingly, TDP-43 in AD interacts with Aβ, inhibiting its fibrillization but potentially worsening overall AD pathology in mouse models [95]. Additionally, TDP-43 concurs with the tau/Aβ aggregates in AD patients and co-deposits in neurons with neurofibrillary tangles (NTFs) and even with senile plaques in the same neurons [17,97]. This suggests a complex three-way interaction between TDP-43, tau, and amyloid in AD. Using immunofluorescence double-labeling of pTDP-43 and phosphorylated tau in PART cases, a study reported co-staining of tau-positive neuropil threads and pTDP-43-positive DNs in the hippocampus, with additional neurons also showing co-localization of tau and pTDP-43 in NCIs [92].

From a clinical standpoint, the presence of TDP-43 alongside tau in both PART and AD is consistently associated with more severe cognitive impairment and faster rates of cognitive decline, compared to cases with tau or amyloid alone [2,3,14,91,96]. TDP-43 co-pathology can contribute to the heterogeneity of AD clinical presentations, potentially making diagnosis more challenging if relying solely on standard AD criteria. For instance, in AD, a TDP-43 pattern resembling FTLD-TDP is linked to a higher frequency of frontotemporal dementia (FTD)-like symptoms such as behavioral and language problems, in addition to the typical amnestic presentation of AD [7,98]. Similarly, limbic-predominant TDP-43 encephalopathy (LATE), which represents the clinical manifestation of limbic-predominant TDP-43 encephalopathy-neuropathologic changes (LATE-NC) and is often associated with PART [99], is characterized by the presence of TDP-43 and can lead to more global cognitive involvement, particularly affecting semantic memory, especially when comorbid with hippocampal sclerosis [16]. The presence or absence of amyloid biomarkers is crucial for differentiating probable PART from AD [100]. However, in amyloid-positive individuals, the co-localization of TDP-43 and tau can further complicate the picture, and the degree to which symptoms are attributable to each pathology is often unclear without specific TDP-43 biomarkers [16]. This further complicates therapeutic trials, given that the presence of LATE-NC may reduce the efficacy of anti-amyloid agents, as the ongoing TDP-43 pathology would continue to drive neurodegeneration [16,100]. Notably, age-related increases in mixed pathology suggest that some concurrent pathologies may represent secondary phenomena rather than primary disease processes [5,59].

### 3.5. Tau in Limbic-Predominant Age-Related TDP-43 Encephalopathy

A significant portion of what is currently referred to as LATE-NC aligns with previously documented patterns of TDP-43 pathology in older adults [101,102,103,104]. The term “LATE” was introduced to facilitate standardized discussions regarding age-related TDP-43 pathology, particularly in limbic structures such as the hippocampus and amygdala [4,105]. It is essential to acknowledge that the fundamental neuropathological features associated with LATE have been well characterized in the earlier TDP-43 literature [96,106,107]. Consequently, findings related to LATE may often represent reclassifications of previous research rather than entirely novel disease concepts. Nonetheless, this framework could be considered instrumental in organizing and comparing research across various studies, particularly in contexts involving comorbid tau or amyloid pathology.

Studies indicate that LATE-NC is associated with an increased burden of pre-tangles and/or NFT in regions like the hippocampus and frontal cortex compared to controls and AD cases without LATE-NC [2,6]. Additionally, AD cases with comorbid LATE-NC show increased brain levels of specific phosphorylated tau epitopes, such as p-tau199 [6]. TDP-43 and tau have the potential to co-localize within the same neurons in limbic regions impacted by LATE-NC [2,8]. This co-localization may be associated with NCIs, DNs, and structures resembling NFTs [7,108]. Additionally, early TDP-43 pathology in LATE-NC can be associated with neurons vulnerable to tau pathology, sometimes referred to as “tangle-associated TDP-43” (also known as TATS) due to its NFT-like morphology and potential co-staining with tau antibodies. It is noteworthy to mention that LATE-NC is also associated with more severe (higher Braak NFT stage) PART [106,109].

When LATE-NC co-exists with ADNC, which includes NFT tau pathology, the cognitive decline is generally more rapid than in patients with either LATE or AD alone [16,91,100]. Moreover, individuals with AD and comorbid LATE may exhibit more profound impairments of episodic memory compared to pure LATE [16,107]. This memory loss is characteristic of limbic/hippocampal-type amnesia [16]. Despite the existence of cases where LATE-NC appears to be the primary driver, the concept of “pure” LATE-NC is challenged by the high frequency of co-pathologies in aging brains. LATE-NC frequently co-exists with other neurodegenerative conditions, most notably ADNC, but also cerebrovascular disease, Lewy body disease, and aging-related tau astrogliopathy (ARTAG) [4,16,18,91]. Although in current practice, LATE-NC can be rendered as pure, regardless of the presence of any number and burden of additional pathologies, as long as there is an absence of low ADNC, this further challenges the notion and confirms a common co-pathology of tau in LATE [18].

## 4. Macro- and Microstructural MRI Findings in TDP and Tau Co-Pathologies

The addition of neuroimaging techniques, such as Magnetic Resonance Imaging (MRI), has increased the adoption in the comprehensive study of mixed neurodegenerative pathologies [110]. Such coexistent pathologies increase the odds of dementia up to almost ten-fold and up to three-fold compared to patients with only one pathology [111]. Interestingly, atypical TDP-43 (star shape) inclusions have also been found in a subset of the elderly population (oldest-old) [112]. The patterns of pathologic comorbidities provide circumstantial evidence of mechanistic interactions (“synergies”) between the pathologies and also suggest common molecular influences [4]. As an example, in LATE-NC, previous studies have found worsening tau aggregation and seeding, potentially enhancing neuronal loss and further degeneration [4]. On the other hand, TDP-43 inclusions have been found in more than half of the cases with ADNC cases, often in a limbic distribution [3,113,114,115], and associated with more severe clinical outcomes than AD alone [6,116,117]. In patient populations with these co-pathologies, MRI studies have found that a high TDP-43 stage was associated with smaller cross-sectional brain volumes, faster rates of brain atrophy, and acceleration of atrophy rates, more than a decade before death, with deceleration occurring closer to death, compared to those with pure AD-NC [118]. Thus, the coexistence expression of these anomalous proteins has been associated with MRI measurements of lower hippocampal volumes and increased rates of atrophy over time, in particular in individuals with AD-NC with a high tau burden [119,120].

The coexistence of TDP-43 and tau pathologies is relatively common, particularly primary PART and ARTAG [59]. As an example, recent studies in patients with PART have found a larger decrease in hippocampal subfield volumes in patients who expressed TDP-43 compared to patients who did not [10].

CBD alone has been fairly widely studied using structural MRI methods [121,122,123]. Overall, neuroimaging studies often show asymmetric cortical and subcortical abnormalities, mainly involving peri-Rolandic and parietal regions and basal ganglia structures [124]. Previous studies have shown that CBD displays a focal atrophy of premotor and supplementary motor areas. In addition, diffusion tensor imaging studies (DTI) have shown that increased gray matter (GM) mean diffusivity (MD) and GM loss were identified bilaterally throughout frontal and temporal lobes, with abnormal diffusivity observed in WM tracts that connect to these regions [125]. CBD can also be presented with TDP-43 pathology inclusions, largely presented in deep GM structures [77,88], which may also modify the clinicopathological features of CBD [87]. As such, studies have found a significant topographical correlation between neuronal TDP-43 cytoplasmic aggregation and neuronal loss in CBD, possibly indicating that TDP-43 protein aberrations in CBD are likely to be associated with neurodegeneration processes [88]. Additional studies have shown positive correlations between the severities of TDP-43 and four-repeat (4R)-tau aggregates in the cervical cord on subjects with PSP and CBD [12]. As a rare form, CBD is also associated with olivopontocerebellar atrophy and TDP-43 pathology (CBD-OPCA) with greater infratentorial tau burden, especially in the pontine base, in CBD-OPCA compared with typical CBD [126]. In isolated cases, structural MRI findings have reported marked atrophy of the pons, midbrain, and cerebellum [127]. In large studies, it has been reported that 5% of PSP patients showed TDP-43 aggregates in the hippocampal regions [128].

**Figure 4:**
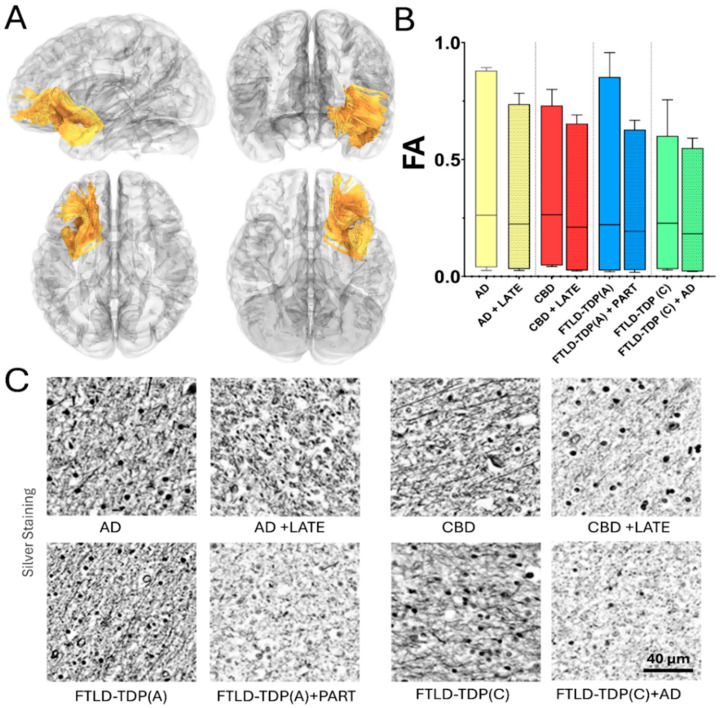
Clinical neuroimaging examples of microstructural white matter changes in a subgroup of illustrative cases with multi-dementia expressing abnormal tau and TDP-43. (**A**) Diffusion tensor imaging-based tractography representations on different views along the left uncinate fasciculi (in yellow). (**B**) Fractional anisotropy (FA) from these tracts where decreased (increased microstructural worsening) and worse in cases expressing simultaneously phosphorylated tau and TDP-43. (**C**) Gallyas-silver staining across representative white matter in the left temporal regions from subjects with premortem neuroimaging displayed in (**B**), showing increased microstructural alterations in neurofilament organization in patients with tau and TDP-43 multi-pathologies. Note that the comparison was conducted based on subjects with equal ADNC scores and comparable premortem imaging, as well as the length of the disease.

In FTLD, sporadic case reports of patients presenting with language and speech impairments with mixed tauopathy (argyrophilic grain disease) and TDP-43 neuropathology revealed MRI findings of left mesial temporal atrophy [129]. In a larger case series, MRI volumetry studies have found that the neuroimaging signature of such mixed cases included more widespread atrophy than that of pure groups. Specifically, cases of mixed CBD with FTLD-TDP showed more prominent asymmetric left-sided atrophy than those of pure CBD [60]. To date, a few studies have been designed to evaluate the combined contribution of this misfolded protein to brain tissue microstructure. Recent microstructural studies have revealed an association between a higher LATE stage with lower white matter fractional anisotropy (FA) in a brain network subset related to stereotypical AD [130,131]. Overall, the field of mixed neuropathology still needs to be further examined by neuroimaging microstructural methods. The addition of diffusion MRI-derived techniques in the study of typical and atypical dementias [132,133,134] could be potentially useful to evaluate the impact of mixed pathology in brain connectomes and possibly determine the individual contribution of each co-pathology, helping in the overall clinical diagnosis (Figure 4).

**Figure 5:**
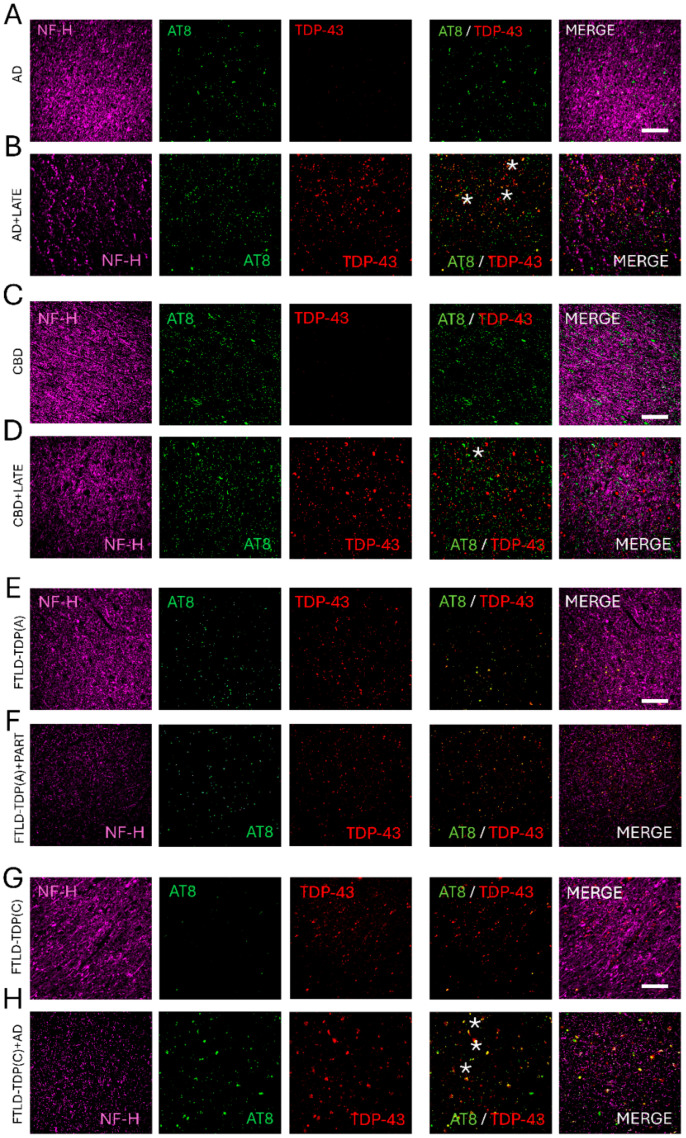
Immunohistochemical patterns of temporal white matter regions by fluorescence confocal microscopy from a short representative set of patients diagnosed with mixed dementias. (**A**) Confocal evaluation of temporal white matter (WM) tracts by neurofilament heavy chain (NF-H) markers (magenta), phosphorylated tau (AT8 in green), and phosphorylated TDP-43 (red) is shown from a patient with AD. Note a decrease in neurofilament content in patients with AD and LATE (**B**). A sample from a subject with CBD (**C**) showed a more preserved WM microstructure than a patient with CBD and LATE (**D**). Similar increasing alterations were seen between FTLD-TDP subjects with the additional presence of PART (**E** vs. **F**), as well as AD (**G** vs. **H**) co-pathologies. Note the increased co-localization between AT8 and TDP-43 (asterisk), which could indicate a coexistent burden mechanism. Scale bar = 20 microns.

It is worth noticing that current efforts are centered on validating the microstructural changes determined using diffusion techniques and different structural and biochemical biomarkers obtained via neuropathological techniques. Although these techniques cannot determine the exact location of each pathological protein, an overall approximation can be noted by their influence on the disruption of white matter connectivity (Figure 5).

## 5. Positron Emission Tomography (PET) Scan

While PET imaging plays an increasingly important role in identifying various pathologies associated with neurodegenerative diseases, including tau, direct in vivo detection of TDP-43 pathology remains challenging [135,136,137,138]. Hence, the role of PET in identifying co-pathologies in mixed dementias is limited.

### 5.1. Tau PET Scan and TDP-43 Detection

Several PET tracers have been developed to image tau pathology, particularly the NFT associated with AD [135,136,139,140,141]. On the other hand, such tau-PET tracer has a limited capacity to represent the histopathological tau burden in non-AD patients, although there are instances where regional uptake correlates with regional tau burden [53]. These include first-generation tracers like 18F-flortaucipir (formerly 18F-AV-1451) and newer second-generation compounds [135,141,142]. Tau PET may help in the differential diagnosis of AD and non-AD dementias, assessing disease severity, and potentially tracking disease progression [135,136,142]. Although a suitable radiotracer that selectively targets TDP-43 for PET imaging has not yet been developed for clinical use, some studies have explored whether existing tau PET radioligands might bind to TDP-43 aggregates due to shared structural properties such as beta-pleated sheets [143,144].

**Figure 6:**
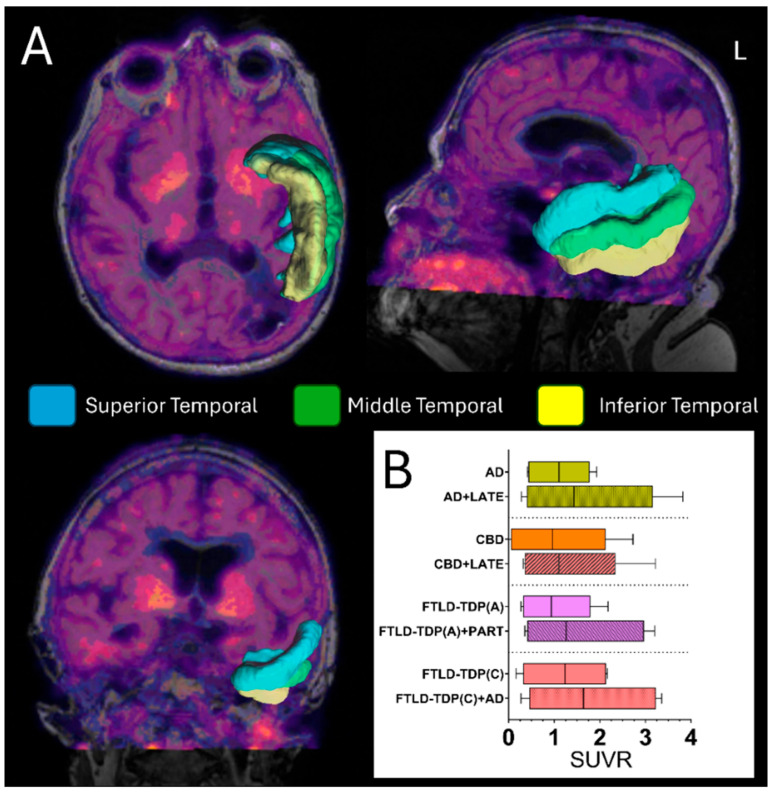
Selected clinical examples to illustrate molecular imaging changes of mixed dementia pathologies by tau-PET imaging. *(***A**) Representative axial, lateral, and coronal views by tau-PET imaging and automatic segmentation from regions of interest (ROIs) including the superior (blue), middle (green), and inferior (yellow) left temporal lobe. (**B**) Quantitative evaluation of the average tau-PET bindings from the combined left temporal ROIs by standardized uptake ratio (SUVR). Considering comparable ADNC scores between subjects with single and dual pathology, we observed a larger amount of tau-deposition in subjects with co-pathologies. This may indicate that the presence of TDP-43 could enhance the deposition by tau-PET bindings in such complex cases.

Few in vitro studies have shown that [3H]MK-6240, [3H]JNJ-067, and [3H]GTP-1 did not bind to TDP-43, while [3H]CBD-2115 showed marginal specific binding that did not consistently correlate with pTDP-43 [143]. Additionally, [3H]flortaucipir did not show a significant correlation with pTDP-43 pathology. However, it showed a trend towards a positive relationship with ALS tau pathology [143]. Moreover, [3H]APN-1607 (18F-Florzolotau) correlated most strongly with amyloid load and did not indicate pTDP-43 pathology [143]. On the other hand, while one in vivo study suggested that 18F-flortaucipir mirrored the expected distribution of TDP-43 pathology in patients with semantic variant primary progressive aphasia (svPPA) [145], another study in older individuals showed that TDP-43 pathology did not affect [18F]-flortaucipir uptake [144]. Hence, while some tau-PET tracers might exhibit off-target binding to TDP-43, this is not consistent across all tracers and may not necessarily represent a true augmentation of the tau-specific signal. The signal in tau-PET would primarily reflect the burden and distribution of tau pathology, but it is not a reliable method for investigating TDP-43 co-occurrence. However, tau-PET could indirectly notice the added burden of TDP-43, unveiling an additional tau accumulation in cases with co-pathologies (Figure 6).

### 5.2. Fluorodeoxyglucose (FDG) PET Scan Significance in Detecting Tau and TDP-43

[18F]-fluorodeoxyglucose (FDG) PET can help in the differential diagnosis of various dementias by identifying specific hypometabolic patterns. In the context of TDP-43 proteinopathies, some studies on ALS have reported frontal hypometabolism and posterior hypermetabolism [146,147]. Although FDG-PET can sometimes reveal patterns suggestive of mixed pathologies, for example, showing features consistent with both PSP and AD [138], it is not specific enough to directly identify TDP-43 pathology or its co-occurrence with tau, even though the presence of both pathologies could potentially lead to more widespread metabolic dysfunction and more hypometabolism signals [137,138,148].

Previous research has indicated that FDG-PET imaging in AD patients with co-existing TDP-43 proteinopathy reveals greater hypometabolism in specific brain regions, such as the medial temporal, frontal superior medial, and frontal supraorbital regions, when compared to AD patients without TDP-43 pathology [138,149]. This supports the idea that the presence of TDP-43 pathology can exacerbate the metabolic deficits typically seen in AD [138,149]. However, FDG-PET patterns are generally not specific enough to differentiate between the contributions of different underlying pathologies, such as tau and TDP-43, and that is because overlapping pathologies make interpretation challenging. Therefore, it is reasonable to consider that the co-occurrence of TDP-43 and tau could lead to more pronounced neurodegenerative effects, potentially resulting in increased hypometabolism on FDG-PET. Nonetheless, it is important to note that FDG-PET is not specific to these individual proteins.

### 5.3. Neuroimaging and Neuromodulation in Untangling Mixed Pathology Networks

The intricate interplay of these co-pathologies results in interconnected network disruptions that traditional PET imaging finds challenging to address. Recent advancements in multi-modal neuroimaging, combined with neuromodulation techniques, offer new avenues for investigating these interactions. By integrating structural and functional MRI with non-invasive brain stimulation (NIBS) protocols, we gain a comprehensive perspective: neuroimaging elucidates network-level correlations, while NIBS elucidates causal relationships between specific circuits and clinical phenotypes [150]. For instance, Cortico-cortical paired associative stimulation (ccPAS) has shown effects on synaptic plasticity that are specific to age and pathology. Notably, in Alzheimer’s disease, there are preserved mechanisms resembling long-term depression (LTD), which stand in contrast to the impaired long-term potentiation (LTP)-like plasticity observed [151]. This differential response pattern may assist in distinguishing primary tauopathy from TDP-43-mediated network dysfunction when analyzed in conjunction with tau-PET findings.

Recent research on cortical excitability (CE) biomarkers offers valuable insights into the detection of co-pathological impacts. CE measurements obtained through transcranial magnetic stimulation (TMS)-EEG protocols indicate altered excitation–inhibition balances that are associated with distinct cognitive resilience profiles [152]. In cases of mixed dementia, cognitive engagement (CE) mapping has the potential to identify early TDP-43-mediated hyperexcitability within limbic networks, even before the emergence of pathology visible through PET imaging. This is particularly relevant in instances where tau-PET reveals atypical distribution patterns. This methodology is consistent with the inverted-U model of the relationship between excitability and cognition, wherein pathological deviations in CE from optimal ranges serve as indicators of impending functional decline [152]. The analysis of the white matter connectome through diffusion MRI, when combined with these functional measures, has the potential to elucidate the “double hit” phenomenon associated with concurrent tau and TDP-43 pathology. Recent studies highlight the significant applications of machine learning in structural MRI, which have successfully differentiated brain patterns associated with schizophrenia [150]. This approach can be adapted for the analysis of mixed dementia subtypes. Future protocols that incorporate tau-PET for mapping primary pathology, CE profiling to evaluate network excitability states, ccPAS for assessing plasticity reserve, and diffusion tensor imaging for examining axonal integrity may facilitate the development of multi-parametric biomarkers.

We must recognize that certain significant limitations persist, particularly regarding the current techniques’ inability to spatially resolve protein-specific contributions within overlapping network disruptions. Nevertheless, the studies reviewed indicate promising translational pathways.

## 6. Summary

In this review, we identified a significant body of research investigating the co-occurrence of TDP-43 and tau proteinopathies across various mixed dementia settings. We investigated clinicopathological studies, in vitro and in vivo models, and biomarker research. Our study indicates a frequent co-occurrence of these two significant proteinopathies in the aging brain and diverse neurodegenerative diseases. These findings indicate a significant overlap of tau and TDP-43 proteinopathies in several neurodegenerative disorders, which suggests a complex interplay that contributes to the diverse clinical presentation and progression rates observed in mixed dementia.

The co-occurrence of tau and TDP-43 appears to be a significant contributor to the clinical heterogeneity seen in mixed dementias [148]. The presence of multiple neuropathologies may influence the rate of cognitive decline and the specific cognitive and behavioral profiles observed in patients [5,18]. For example, the presence of mixed pathologies, rather than a single one, might contribute to hippocampal degeneration in some individuals with amnestic presentations [18].

The concept of the “double-punch scenario” suggests potential synergistic or interactive effects between tau and TDP-43 proteinopathies. This could exacerbate neuronal dysfunction and accelerate neurodegeneration [95,139,153]. This co-occurrence of both proteinopathies has a more damaging impact than a single proteinopathy.

Further research is essential to fully understand the intricate interplay between tau and TDP-43 in the pathogenesis of mixed dementia. This includes improvements in diagnostic tools, further understanding of the molecular mechanisms of their interaction, and the potential targeted therapeutic strategies that address this complex relationship.

## 7. Conclusions

The current pathological criteria and study methodologies, particularly the reliance on traditional markers for mature NFTs rather than markers for earlier tau pathology like PHF-1, may systematically underestimate tau pathology when it coexists with TDP-43. Furthermore, limited sampling protocols and regional analysis patterns often miss concurrent pathologies because TDP-43 in mixed cases can have restricted distributions. Given that, we advocate for unified neuropathological protocols that comprehensively assess both pathologies across all brain regions to overcome these biases.

Additionally, the clinical presentation of mixed pathologies can result in an intricate or overlapping array of symptoms that do not precisely align with established subtypes, thereby hindering the clinical identification of the specific underlying proteinopathies. Therefore, we emphasize that relying primarily on clinical diagnostic criteria for classification is impractical and often underrepresents the true prevalence of mixed pathologies. We also emphasize the critical role of precision medicine approaches, which will be essential for future therapeutic interventions. Treatments must be customized not only to address primary pathologies but also to accommodate specific combinations of pathologies.

Moreover, it is important to note that while tau-PET tracers have proven useful for assessing tau, their capacity to accurately represent the histopathological tau burden in non-AD patients remains limited. Furthermore, these methods do not provide reliable means for directly investigating the co-occurrence of TDP-43, despite the possibility of some off-target binding. Similarly, FDG-PET demonstrates increased hypometabolism in mixed cases; however, it lacks the specificity required to differentiate the contributions of individual pathologies such as tau and TDP-43.

A comprehensive mechanistic understanding of the “pathologic synergy” that extends beyond mere co-occurrence is essential. While co-occurrence is a common observation, the intricate molecular interactions are still being elucidated. Therefore, it is imperative to conduct more focused research not only on co-existence but also on the complex bidirectional interactions and specific molecular pathways. This approach will enhance our understanding and enable more effective interventions in this multifaceted interplay.

This review highlights the prevalence and significance of tau and TDP-43 co-pathology in the context of mixed dementias. Recognizing the double-punch scenario is crucial for researchers and healthcare providers to improve our understanding, diagnosis, and management of these challenging neurodegenerative disorders. Increased focus on developing multimodal biomarkers and investigating the synergistic effects of these pathologies is essential for advancing the field.

## Data Availability

Not applicable.

## References

[1] Gefen T., Ahmadian S.S., Mao Q., Kim G., Seckin M., Bonakdarpour B., Ramos E.M., Coppola G., Rademakers R., Rogalski E. (2018). Combined Pathologies in FTLD-TDP Types A and C. J. Neuropathol. Exp. Neurol..

[2] Latimer C.S., Liachko N.F. (2021). Tau and TDP-43 synergy: A novel therapeutic target for sporadic late-onset Alzheimer’s disease. Geroscience.

[3] Meneses A., Koga S., O’Leary J., Dickson D.W., Bu G., Zhao N. (2021). TDP-43 Pathology in Alzheimer’s Disease. Mol. Neurodegener..

[4] Nelson P.T., Fardo D.W., Wu X., Aung K.Z., Cykowski M.D., Katsumata Y. (2024). Limbic-predominant age-related TDP-43 encephalopathy (LATE-NC): Co-pathologies and genetic risk factors provide clues about pathogenesis. J. Neuropathol. Exp. Neurol..

[5] Pennington C., Marini L., Coulthard E., Love S. (2020). Mixed neuropathology in frontotemporal lobar degeneration. Amyotroph. Lateral Scler. Front. Degener..

[6] Tome S.O., Tsaka G., Ronisz A., Ospitalieri S., Gawor K., Gomes L.A., Otto M., von Arnim C.A.F., Van Damme P., Van Den Bosch L. (2023). TDP-43 pathology is associated with increased tau burdens and seeding. Mol. Neurodegener..

[7] Tome S.O., Vandenberghe R., Ospitalieri S., Van Schoor E., Tousseyn T., Otto M., von Arnim C.A.F., Thal D.R. (2020). Distinct molecular patterns of TDP-43 pathology in Alzheimer’s disease: Relationship with clinical phenotypes. Acta Neuropathol. Commun..

[8] Josephs K.A., Murray M.E., Tosakulwong N., Weigand S.D., Knopman D.S., Petersen R.C., Jack C.R., Whitwell J.L., Dickson D.W. (2019). Brain atrophy in primary age-related tauopathy is linked to transactive response DNA-binding protein of 43 kDa. Alzheimers Dement..

[9] Riku Y., Yoshida M., Iwasaki Y., Sobue G., Katsuno M., Ishigaki S. (2022). TDP-43 Proteinopathy and Tauopathy: Do They Have Pathomechanistic Links?. Int. J. Mol. Sci..

[10] Youssef H., Gatto R.G., Pham N.T.T., Petersen R.C., Machulda M.M., Reichard R.R., Dickson D.W., Jack C.R., Whitwell J.L., Josephs K.A. (2024). TDP-43 Is Associated with Subiculum and Cornu Ammonis 1 Hippocampal Subfield Atrophy in Primary Age-Related Tauopathy. J. Alzheimers Dis..

[11] McAleese K.E., Walker L., Erskine D., Johnson M., Koss D., Thomas A.J., Attems J. (2020). Concomitant LATE-NC in Alzheimer’s disease is not associated with increased tau or amyloid-beta pathological burden. Neuropathol. Appl. Neurobiol..

[12] Riku Y., Iwasaki Y., Ishigaki S., Akagi A., Hasegawa M., Nishioka K., Li Y., Riku M., Ikeuchi T., Fujioka Y. (2022). Motor neuron TDP-43 proteinopathy in progressive supranuclear palsy and corticobasal degeneration. Brain.

[13] Tome S.O., Gawor K., Thal D.R. (2024). LATE-NC in Alzheimer’s disease: Molecular aspects and synergies. Brain Pathol..

[14] Josephs K.A., Murray M.E., Tosakulwong N., Whitwell J.L., Knopman D.S., Machulda M.M., Weigand S.D., Boeve B.F., Kantarci K., Petrucelli L. (2017). Tau aggregation influences cognition and hippocampal atrophy in the absence of beta-amyloid: A clinico-imaging-pathological study of primary age-related tauopathy (PART). Acta Neuropathol..

[15] Josephs K.A., Whitwell J.L., Knopman D.S., Hu W.T., Stroh D.A., Baker M., Rademakers R., Boeve B.F., Parisi J.E., Smith G.E. (2008). Abnormal TDP-43 immunoreactivity in AD modifies clinicopathologic and radiologic phenotype. Neurology.

[16] Nelson P.T., Schneider J.A., Jicha G.A., Duong M.T., Wolk D.A. (2023). When Alzheimer’s is LATE: Why Does it Matter?. Ann. Neurol..

[17] Jiang L.L., Zhang X.L., Hu H.Y. (2024). Co-Aggregation of TDP-43 with Other Pathogenic Proteins and Their Co-Pathologies in Neurodegenerative Diseases. Int. J. Mol. Sci..

[18] Youssef H., Gatto R.G., Pham N.T.T., Jones D., Petersen R.C., Machulda M.M., Whitwell J.L., Josephs K.A. (2025). Multiple Neuropathologies Underly Hippocampal Subfield Atrophy in a Case With a Slowly Progressive Amnestic Syndrome: Challenging the Notion of Pure LATE-NC. Neuropathology.

[19] Dulski J., Cerquera-Cleves C., Milanowski L., Kidd A., Sitek E.J., Strongosky A., Vanegas Monroy A.M., Dickson D.W., Ross O.A., Pentela-Nowicka J. (2021). Clinical, pathological and genetic characteristics of Perry disease-new cases and literature review. Eur. J. Neurol..

[20] Mishima T., Fujioka S., Tomiyama H., Yabe I., Kurisaki R., Fujii N., Neshige R., Ross O.A., Farrer M.J., Dickson D.W. (2018). Establishing diagnostic criteria for Perry syndrome. J. Neurol. Neurosurg. Psychiatry.

[21] Baloh R.H. (2011). TDP-43: The relationship between protein aggregation and neurodegeneration in amyotrophic lateral sclerosis and frontotemporal lobar degeneration. Febs J..

[22] Tollervey J.R., Curk T., Rogelj B., Briese M., Cereda M., Kayikci M., Konig J., Hortobagyi T., Nishimura A.L., Zupunski V. (2011). Characterizing the RNA targets and position-dependent splicing regulation by TDP-43. Nat. Neurosci..

[23] Buratti E., Baralle F.E. (2010). The multiple roles of TDP-43 in pre-mRNA processing and gene expression regulation. RNA Biol..

[24] Ishiguro T., Sato N., Ueyama M., Fujikake N., Sellier C., Kanegami A., Tokuda E., Zamiri B., Gall-Duncan T., Mirceta M. (2017). Regulatory Role of RNA Chaperone TDP-43 for RNA Misfolding and Repeat-Associated Translation in SCA31. Neuron.

[25] Ma X., Ying Y., Xie H., Liu X., Wang X., Li J. (2021). The Regulatory Role of RNA Metabolism Regulator TDP-43 in Human Cancer. Front. Oncol..

[26] Freibaum B.D., Chitta R.K., High A.A., Taylor J.P. (2010). Global analysis of TDP-43 interacting proteins reveals strong association with RNA splicing and translation machinery. J. Proteome Res..

[27] Lalmansingh A.S., Urekar C.J., Reddi P.P. (2011). TDP-43 is a transcriptional repressor: The testis-specific mouse acrv1 gene is a TDP-43 target in vivo. J. Biol. Chem..

[28] Dutta K., Thammisetty S.S., Boutej H., Bareil C., Julien J.P. (2020). Mitigation of ALS Pathology by Neuron-Specific Inhibition of Nuclear Factor Kappa B Signaling. J. Neurosci..

[29] Chhangani D., Martin-Pena A., Rincon-Limas D.E. (2021). Molecular, functional, and pathological aspects of TDP-43 fragmentation. iScience.

[30] Fang Y.S., Tsai K.J., Chang Y.J., Kao P., Woods R., Kuo P.H., Wu C.C., Liao J.Y., Chou S.C., Lin V. (2014). Full-length TDP-43 forms toxic amyloid oligomers that are present in frontotemporal lobar dementia-TDP patients. Nat. Commun..

[31] Liachko N.F., Guthrie C.R., Kraemer B.C. (2010). Phosphorylation promotes neurotoxicity in a Caenorhabditis elegans model of TDP-43 proteinopathy. J. Neurosci..

[32] Neumann M., Lee E.B., Mackenzie I.R. (2021). Frontotemporal Lobar Degeneration TDP-43-Immunoreactive Pathological Subtypes: Clinical and Mechanistic Significance. Adv. Exp. Med. Biol..

[33] Kawakami I., Arai T., Hasegawa M. (2019). The basis of clinicopathological heterogeneity in TDP-43 proteinopathy. Acta Neuropathol..

[34] Barbier P., Zejneli O., Martinho M., Lasorsa A., Belle V., Smet-Nocca C., Tsvetkov P.O., Devred F., Landrieu I. (2019). Role of Tau as a Microtubule-Associated Protein: Structural and Functional Aspects. Front. Aging Neurosci..

[35] Di Lorenzo D. (2024). Tau Protein and Tauopathies: Exploring Tau Protein–Protein and Microtubule Interactions, Cross-Interactions and Therapeutic Strategies. ChemMedChem.

[36] Avila J., Lucas J.J., Perez M., Hernandez F. (2004). Role of tau protein in both physiological and pathological conditions. Physiol. Rev..

[37] Regan P., Mitchell S.J., Kim S.C., Lee Y., Yi J.H., Barbati S.A., Shaw C., Cho K. (2021). Regulation of Synapse Weakening through Interactions of the Microtubule Associated Protein Tau with PACSIN1. J. Neurosci..

[38] Robbins M., Clayton E., Kaminski Schierle G.S. (2021). Synaptic tau: A pathological or physiological phenomenon?. Acta Neuropathol. Commun..

[39] Kanaan N.M. (2024). Tau here, tau there, tau almost everywhere: Clarifying the distribution of tau in the adult CNS. Cytoskelet..

[40] Younas N., Saleem T., Younas A., Zerr I. (2023). Nuclear face of Tau: An inside player in neurodegeneration. Acta Neuropathol. Commun..

[41] Gil L., Federico C., Pinedo F., Bruno F., Rebolledo A.B., Montoya J.J., Olazabal I.M., Ferrer I., Saccone S. (2017). Aging dependent effect of nuclear tau. Brain Res..

[42] Tang X., Jiao L., Zheng M., Yan Y., Nie Q., Wu T., Wan X., Zhang G., Li Y., Wu S. (2018). Tau Deficiency Down-Regulated Transcription Factor Orthodenticle Homeobox 2 Expression in the Dopaminergic Neurons in Ventral Tegmental Area and Caused No Obvious Motor Deficits in Mice. Neuroscience.

[43] Bukar Maina M., Al-Hilaly Y.K., Serpell L.C. (2016). Nuclear Tau and Its Potential Role in Alzheimer’s Disease. Biomolecules.

[44] Grundke-Iqbal I., Iqbal K., Quinlan M., Tung Y.C., Zaidi M.S., Wisniewski H.M. (1986). Microtubule-associated protein tau. A component of Alzheimer paired helical filaments. J. Biol. Chem..

[45] Corsi A., Bombieri C., Valenti M.T., Romanelli M.G. (2022). Tau Isoforms: Gaining Insight into MAPT Alternative Splicing. Int. J. Mol. Sci..

[46] Liu F., Gong C.X. (2008). Tau exon 10 alternative splicing and tauopathies. Mol. Neurodegener..

[47] Strang K.H., Golde T.E., Giasson B.I. (2019). MAPT mutations, tauopathy, and mechanisms of neurodegeneration. Lab. Investig..

[48] Andreadis A., Brown W.M., Kosik K.S. (1992). Structure and novel exons of the human tau gene. Biochemistry.

[49] Ferrer I., López-González I., Carmona M., Arregui L., Dalfó E., Torrejón-Escribano B., Diehl R., Kovacs G.G. (2014). Glial and neuronal tau pathology in tauopathies: Characterization of disease-specific phenotypes and tau pathology progression. J. Neuropathol. Exp. Neurol..

[50] Kahlson M.A., Colodner K.J. (2015). Glial Tau Pathology in Tauopathies: Functional Consequences. J. Exp. Neurosci..

[51] Eltom K., Mothes T., Libard S., Ingelsson M., Erlandsson A. (2024). Astrocytic accumulation of tau fibrils isolated from Alzheimer’s disease brains induces inflammation, cell-to-cell propagation and neuronal impairment. Acta Neuropathol. Commun..

[52] Dickson D.W., Kouri N., Murray M.E., Josephs K.A. (2011). Neuropathology of frontotemporal lobar degeneration-tau (FTLD-tau). J. Mol. Neurosci..

[53] Gatto R.G., Carlos A.F., Reichard R.R., Lowe V.J., Whitwell J.L., Josephs K.A. (2023). Comparative assessment of regional tau distribution by Tau-PET and Post-mortem neuropathology in a representative set of Alzheimer’s & frontotemporal lobar degeneration patients. PLoS ONE.

[54] Gatto R.G., Hossam Y., Reichard R.R., Lowe V.J., Whitwell J.L., Josephs K.A. (2024). Microscopy assessment of a fluorescence [(18)F] flortaucipir analog (T726) shows neuropathological overlap with 3R and 4R tau lesions. Alzheimers Dement..

[55] Chatterjee M., Ozdemir S., Fritz C., Mobius W., Kleineidam L., Mandelkow E., Biernat J., Dogdu C., Peters O., Cosma N.C. (2024). Plasma extracellular vesicle tau and TDP-43 as diagnostic biomarkers in FTD and ALS. Nat. Med..

[56] Chornenkyy Y., Fardo D.W., Nelson P.T. (2019). Tau and TDP-43 proteinopathies: Kindred pathologic cascades and genetic pleiotropy. Lab. Invest..

[57] Taylor L.M., McMillan P.J., Liachko N.F., Strovas T.J., Ghetti B., Bird T.D., Keene C.D., Kraemer B.C. (2018). Pathological phosphorylation of tau and TDP-43 by TTBK1 and TTBK2 drives neurodegeneration. Mol. Neurodegener..

[58] Liachko N.F., McMillan P.J., Strovas T.J., Loomis E., Greenup L., Murrell J.R., Ghetti B., Raskind M.A., Montine T.J., Bird T.D. (2014). The tau tubulin kinases TTBK1/2 promote accumulation of pathological TDP-43. PLoS Genet..

[59] Koga S., Zhou X., Murakami A., Fernandez De Castro C., Baker M.C., Rademakers R., Dickson D.W. (2022). Concurrent tau pathologies in frontotemporal lobar degeneration with TDP-43 pathology. Neuropathol. Appl. Neurobiol..

[60] Kim E.J., Brown J.A., Deng J., Hwang J.L., Spina S., Miller Z.A., DeMay M.G., Valcour V., Karydas A., Ramos E.M. (2018). Mixed TDP-43 proteinopathy and tauopathy in frontotemporal lobar degeneration: Nine case series. J. Neurol..

[61] Robinson A.C., Thompson J.C., Weedon L., Rollinson S., Pickering-Brown S., Snowden J.S., Davidson Y.S., Mann D.M. (2014). No interaction between tau and TDP-43 pathologies in either frontotemporal lobar degeneration or motor neurone disease. Neuropathol. Appl. Neurobiol..

[62] Jo M., Lee S., Jeon Y.-M., Kim S., Kwon Y., Kim H.-J. (2020). The role of TDP-43 propagation in neurodegenerative diseases: Integrating insights from clinical and experimental studies. Exp. Mol. Med..

[63] Gu J., Wu F., Xu W., Shi J., Hu W., Jin N., Qian W., Wang X., Iqbal K., Gong C.X. (2017). TDP-43 suppresses tau expression via promoting its mRNA instability. Nucleic Acids Res..

[64] Gu J., Chen F., Iqbal K., Gong C.X., Wang X., Liu F. (2017). Transactive response DNA-binding protein 43 (TDP-43) regulates alternative splicing of tau exon 10: Implications for the pathogenesis of tauopathies. J. Biol. Chem..

[65] Dang M., Wu L., Zhang X. (2025). Structural insights and milestones in TDP-43 research: A comprehensive review of its pathological and therapeutic advances. Int. J. Biol. Macromol..

[66] Montalbano M., McAllen S., Cascio F.L., Sengupta U., Garcia S., Bhatt N., Ellsworth A., Heidelman E.A., Johnson O.D., Doskocil S. (2020). TDP-43 and Tau Oligomers in Alzheimer’s Disease, Amyotrophic Lateral Sclerosis, and Frontotemporal Dementia. Neurobiol. Dis..

[67] Nelson P.T., Abner E.L., Patel E., Anderson S., Wilcock D.M., Kryscio R.J., Van Eldik L.J., Jicha G.A., Gal Z., Nelson R.S. (2018). The Amygdala as a Locus of Pathologic Misfolding in Neurodegenerative Diseases. J. Neuropathol. Exp. Neurol..

[68] Kapasi A., DeCarli C., Schneider J.A. (2017). Impact of multiple pathologies on the threshold for clinically overt dementia. Acta Neuropathol..

[69] Thomas D.X., Bajaj S., McRae-McKee K., Hadjichrysanthou C., Anderson R.M., Collinge J. (2020). Association of TDP-43 proteinopathy, cerebral amyloid angiopathy, and Lewy bodies with cognitive impairment in individuals with or without Alzheimer’s disease neuropathology. Sci. Rep..

[70] Kawas C.H., Kim R.C., Sonnen J.A., Bullain S.S., Trieu T., Corrada M.M. (2015). Multiple pathologies are common and related to dementia in the oldest-old: The 90 + Study. Neurology.

[71] Hodges J.R., Davies R.R., Xuereb J.H., Casey B., Broe M., Bak T.H., Kril J.J., Halliday G.M. (2004). Clinicopathological correlates in frontotemporal dementia. Ann. Neurol..

[72] Kertesz A., McMonagle P., Blair M., Davidson W., Munoz D.G. (2005). The evolution and pathology of frontotemporal dementia. Brain.

[73] Forman M.S., Farmer J., Johnson J.K., Clark C.M., Arnold S.E., Coslett H.B., Chatterjee A., Hurtig H.I., Karlawish J.H., Rosen H.J. (2006). Frontotemporal dementia: Clinicopathological correlations. Ann. Neurol..

[74] Snowden J., Neary D., Mann D. (2007). Frontotemporal lobar degeneration: Clinical and pathological relationships. Acta Neuropathol..

[75] Torres A.K., Jara C., Olesen M.A., Tapia-Rojas C. (2021). Pathologically phosphorylated tau at S396/404 (PHF-1) is accumulated inside of hippocampal synaptic mitochondria of aged Wild-type mice. Sci. Rep..

[76] Mammeri N.E., Dregni A.J., Duan P., Hong M. (2024). Structures of AT8 and PHF1 phosphomimetic tau: Insights into the posttranslational modification code of tau aggregation. Proc. Natl. Acad. Sci. USA.

[77] Uryu K., Nakashima-Yasuda H., Forman M.S., Kwong L.K., Clark C.M., Grossman M., Miller B.L., Kretzschmar H.A., Lee V.M., Trojanowski J.Q. (2008). Concomitant TAR-DNA-binding protein 43 pathology is present in Alzheimer disease and corticobasal degeneration but not in other tauopathies. J. Neuropathol. Exp. Neurol..

[78] Wisse L.E.M., Wuestefeld A., Murray M.E., Jagust W., La Joie R. (2025). Role of tau versus TDP-43 pathology on medial temporal lobe atrophy in aging and Alzheimer’s disease. Alzheimer’s Dement..

[79] Brenowitz W.D., Hubbard R.A., Keene C.D., Hawes S.E., Longstreth W.T., Woltjer R.L., Kukull W.A. (2017). Mixed neuropathologies and estimated rates of clinical progression in a large autopsy sample. Alzheimers Dement..

[80] Armstrong R.A. (2016). Survival in the pre-senile dementia frontotemporal lobar degeneration with TDP-43 proteinopathy: Effects of genetic, demographic and neuropathological variables. Folia Neuropathol..

[81] Perry D.C., Brown J.A., Possin K.L., Datta S., Trujillo A., Radke A., Karydas A., Kornak J., Sias A.C., Rabinovici G.D. (2017). Clinicopathological correlations in behavioural variant frontotemporal dementia. Brain.

[82] Koga S., Murakami A., Soto-Beasley A.I., Walton R.L., Baker M.C., Castanedes-Casey M., Josephs K.A., Ross O.A., Dickson D.W. (2023). Publisher Correction to: Diffuse argyrophilic grain disease with TDP-43 proteinopathy and neuronal intermediate filament inclusion disease: FTLD with mixed tau, TDP-43 and FUS pathologies. Acta Neuropathol. Commun..

[83] Arnold S.J., Dugger B.N., Beach T.G. (2013). TDP-43 deposition in prospectively followed, cognitively normal elderly individuals: Correlation with argyrophilic grains but not other concomitant pathologies. Acta Neuropathol..

[84] Ferrer I., Santpere G., van Leeuwen F.W. (2008). Argyrophilic grain disease. Brain.

[85] Koga S., Murakami A., Martin N.B., Dickson D.W. (2023). The frequency and distribution of TDP-43 pathology in argyrophilic grain disease. J. Neuropathol. Exp. Neurol..

[86] Fujishiro H., Uchikado H., Arai T., Hasegawa M., Akiyama H., Yokota O., Tsuchiya K., Togo T., Iseki E., Hirayasu Y. (2009). Accumulation of phosphorylated TDP-43 in brains of patients with argyrophilic grain disease. Acta Neuropathol..

[87] Koga S., Kouri N., Walton R.L., Ebbert M.T.W., Josephs K.A., Litvan I., Graff-Radford N., Ahlskog J.E., Uitti R.J., van Gerpen J.A. (2018). Corticobasal degeneration with TDP-43 pathology presenting with progressive supranuclear palsy syndrome: A distinct clinicopathologic subtype. Acta Neuropathol..

[88] Sainouchi M., Tada M., Fitrah Y.A., Hara N., Tanaka K., Idezuka J., Aida I., Nakajima T., Miyashita A., Akazawa K. (2022). Brain TDP-43 pathology in corticobasal degeneration: Topographical correlation with neuronal loss. Neuropathol. Appl. Neurobiol..

[89] Tomenaga T., Minatani S., Namba H., Takeda A., Yoshizaki T., Kawabe J., Keyoumu N., Morino H., Higuchi M., Matsubara T. (2025). An autopsy case of type A FTLD-TDP with a GRN mutation presenting with the logopenic variant of primary progressive aphasia at onset and with corticobasal syndrome subsequently. Neuropathology.

[90] Tando S., Kasai T., Mizuta I., Takahashi H., Yaoi T., Saito K., Hojo T., Mizuno T., Hasegawa M., Itoh K. (2021). An autopsy case of corticobasal syndrome due to asymmetric degeneration of the motor cortex and substantia nigra with TDP-43 proteinopathy, associated with Alzheimer’s disease pathology. Neuropathology.

[91] Smirnov D.S., Salmon D.P., Galasko D., Edland S.D., Pizzo D.P., Goodwill V., Hiniker A. (2022). TDP-43 Pathology Exacerbates Cognitive Decline in Primary Age-Related Tauopathy. Ann. Neurol..

[92] Zhang X., Sun B., Wang X., Lu H., Shao F., Rozemuller A.J.M., Liang H., Liu C., Chen J., Huang M. (2019). Phosphorylated TDP-43 Staging of Primary Age-Related Tauopathy. Neurosci. Bull..

[93] Glashutter M., Wijesinghe P., Matsubara J.A. (2025). TDP-43 as a potential retinal biomarker for neurodegenerative diseases. Front. Neurosci..

[94] Kim D., Kim H.S., Choi S.M., Kim B.C., Lee M.C., Lee K.H., Lee J.H. (2019). Primary Age-Related Tauopathy: An Elderly Brain Pathology Frequently Encountered during Autopsy. J. Pathol. Transl. Med..

[95] Shih Y.H., Tu L.H., Chang T.Y., Ganesan K., Chang W.W., Chang P.S., Fang Y.S., Lin Y.T., Jin L.W., Chen Y.R. (2020). TDP-43 interacts with amyloid-beta, inhibits fibrillization, and worsens pathology in a model of Alzheimer’s disease. Nat. Commun..

[96] Josephs K.A., Murray M.E., Whitwell J.L., Parisi J.E., Petrucelli L., Jack C.R., Petersen R.C., Dickson D.W. (2014). Staging TDP-43 pathology in Alzheimer’s disease. Acta Neuropathol..

[97] Carlos A.F., Koga S., Graff-Radford N.R., Baker M.C., Rademakers R., Ross O.A., Dickson D.W., Josephs K.A. (2024). Senile plaque-associated transactive response DNA-binding protein 43 in Alzheimer’s disease: A case report spanning 16 years of memory loss. Neuropathology.

[98] Huang W., Zhou Y., Tu L., Ba Z., Huang J., Huang N., Luo Y. (2020). TDP-43: From Alzheimer’s Disease to Limbic-Predominant Age-Related TDP-43 Encephalopathy. Front. Mol. Neurosci..

[99] Zhang L., Chen Y., Liu M., Wang Y., Peng G. (2020). TDP-43 and Limbic-Predominant Age-Related TDP-43 Encephalopathy. Front. Aging Neurosci..

[100] Wolk D.A., Nelson P.T., Apostolova L., Arfanakis K., Boyle P.A., Carlsson C.M., Corriveau-Lecavalier N., Dacks P., Dickerson B.C., Domoto-Reilly K. (2025). Clinical criteria for limbic-predominant age-related TDP-43 encephalopathy. Alzheimers Dement..

[101] Nag S., Yu L., Capuano A.W., Wilson R.S., Leurgans S.E., Bennett D.A., Schneider J.A. (2015). Hippocampal sclerosis and TDP-43 pathology in aging and Alzheimer disease. Ann. Neurol..

[102] Wilson A.C., Dugger B.N., Dickson D.W., Wang D.S. (2011). TDP-43 in aging and Alzheimer’s disease—A review. Int. J. Clin. Exp. Pathol..

[103] Hokkanen S.R.K., Hunter S., Polvikoski T.M., Keage H.A.D., Minett T., Matthews F.E., Brayne C., Mrc C., Group C.C.S. (2018). Hippocampal sclerosis, hippocampal neuron loss patterns and TDP-43 in the aged population. Brain Pathol..

[104] Josephs K.A., Mackenzie I., Frosch M.P., Bigio E.H., Neumann M., Arai T., Dugger B.N., Ghetti B., Grossman M., Hasegawa M. (2019). LATE to the PART-y. Brain.

[105] Nelson P.T., Dickson D.W., Trojanowski J.Q., Jack C.R., Boyle P.A., Arfanakis K., Rademakers R., Alafuzoff I., Attems J., Brayne C. (2019). Limbic-predominant age-related TDP-43 encephalopathy (LATE): Consensus working group report. Brain.

[106] Amador-Ortiz C., Lin W.L., Ahmed Z., Personett D., Davies P., Duara R., Graff-Radford N.R., Hutton M.L., Dickson D.W. (2007). TDP-43 immunoreactivity in hippocampal sclerosis and Alzheimer’s disease. Ann. Neurol..

[107] Josephs K.A., Whitwell J.L., Weigand S.D., Murray M.E., Tosakulwong N., Liesinger A.M., Petrucelli L., Senjem M.L., Knopman D.S., Boeve B.F. (2014). TDP-43 is a key player in the clinical features associated with Alzheimer’s disease. Acta Neuropathol..

[108] Llamas-Rodriguez J., Oltmer J., Marshall M., Champion S., Frosch M.P., Augustinack J.C. (2023). TDP-43 and tau concurrence in the entorhinal subfields in primary age-related tauopathy and preclinical Alzheimer’s disease. Brain Pathol..

[109] Josephs K.A., Koga S., Tosakulwong N., Weigand S.D., Nha Pham T.T., Baker M., Whitwell J.L., Rademakers R., Petrucelli L., Dickson D.W. (2023). Molecular fragment characteristics and distribution of tangle associated TDP-43 (TATs) and other TDP-43 lesions in Alzheimer’s disease. Free Neuropathol..

[110] Tan C.H., Hilal S., Xu X., Vrooman H., Cheng C.Y., Wong T.Y., Venketasubramanian N., Chen C. (2020). MRI Markers of Mixed Pathology and Cognitive Impairment in Multiethnic Asians. J. Alzheimers Dis..

[111] Schneider J.A., Arvanitakis Z., Bang W., Bennett D.A. (2007). Mixed brain pathologies account for most dementia cases in community-dwelling older persons. Neurology.

[112] Carlos A.F., Sekiya H., Koga S., Gatto R.G., Casey M.C., Pham N.T.T., Sintini I., Machulda M.M., Jack C.R., Lowe V.J. (2023). Clinicopathologic features of a novel star-shaped transactive response DNA-binding protein 43 (TDP-43) pathology in the oldest old. J. Neuropathol. Exp. Neurol..

[113] Hu W.T., Josephs K.A., Knopman D.S., Boeve B.F., Dickson D.W., Petersen R.C., Parisi J.E. (2008). Temporal lobar predominance of TDP-43 neuronal cytoplasmic inclusions in Alzheimer disease. Acta Neuropathol..

[114] Kadokura A., Yamazaki T., Lemere C.A., Takatama M., Okamoto K. (2009). Regional distribution of TDP-43 inclusions in Alzheimer disease (AD) brains: Their relation to AD common pathology. Neuropathology.

[115] Nelson P.T., Brayne C., Flanagan M.E., Abner E.L., Agrawal S., Attems J., Castellani R.J., Corrada M.M., Cykowski M.D., Di J. (2022). Frequency of LATE neuropathologic change across the spectrum of Alzheimer’s disease neuropathology: Combined data from 13 community-based or population-based autopsy cohorts. Acta Neuropathol..

[116] Hiya S., Maldonado-Díaz C., Walker J.M., Richardson T.E. (2023). Cognitive symptoms progress with limbic-predominant age-related TDP-43 encephalopathy stage and co-occurrence with Alzheimer disease. J. Neuropathol. Exp. Neurol..

[117] Butler Pagnotti R.M., Pudumjee S.B., Cross C.L., Miller J.B. (2023). Cognitive and Clinical Characteristics of Patients With Limbic-Predominant Age-Related TDP-43 Encephalopathy. Neurology.

[118] Josephs K.A., Martin P.R., Weigand S.D., Tosakulwong N., Buciuc M., Murray M.E., Petrucelli L., Senjem M.L., Spychalla A.J., Knopman D.S. (2020). Protein contributions to brain atrophy acceleration in Alzheimer’s disease and primary age-related tauopathy. Brain.

[119] Buciuc M., Wennberg A.M., Weigand S.D., Murray M.E., Senjem M.L., Spychalla A.J., Boeve B.F., Knopman D.S., Jack C.R., Kantarci K. (2020). Effect Modifiers of TDP-43-Associated Hippocampal Atrophy Rates in Patients with Alzheimer’s Disease Neuropathological Changes. J. Alzheimers Dis..

[120] Josephs K.A., Dickson D.W., Tosakulwong N., Weigand S.D., Murray M.E., Petrucelli L., Liesinger A.M., Senjem M.L., Spychalla A.J., Knopman D.S. (2017). Rates of hippocampal atrophy and presence of post-mortem TDP-43 in patients with Alzheimer’s disease: A longitudinal retrospective study. Lancet Neurol..

[121] Boelmans K., Kaufmann J., Bodammer N., Ebersbach G., Behlau G., Heinze H.J., Niehaus L. (2009). Involvement of motor pathways in corticobasal syndrome detected by diffusion tensor tractography. Mov. Disord..

[122] Josephs K.A., Whitwell J.L., Boeve B.F., Knopman D.S., Petersen R.C., Hu W.T., Parisi J.E., Dickson D.W., Jack C.R. (2010). Anatomical differences between CBS-corticobasal degeneration and CBS-Alzheimer’s disease. Mov. Disord..

[123] Constantinides V.C., Paraskevas G.P., Paraskevas P.G., Stefanis L., Kapaki E. (2019). Corticobasal degeneration and corticobasal syndrome: A review. Clin. Park. Relat. Disord..

[124] Di Stasio F., Suppa A., Marsili L., Upadhyay N., Asci F., Bologna M., Colosimo C., Fabbrini G., Pantano P., Berardelli A. (2019). Corticobasal syndrome: Neuroimaging and neurophysiological advances. Eur. J. Neurol..

[125] Whitwell J.L., Avula R., Senjem M.L., Kantarci K., Weigand S.D., Samikoglu A., Edmonson H.A., Vemuri P., Knopman D.S., Boeve B.F. (2010). Gray and white matter water diffusion in the syndromic variants of frontotemporal dementia. Neurology.

[126] Kouri N., Oshima K., Takahashi M., Murray M.E., Ahmed Z., Parisi J.E., Yen S.-H.C., Dickson D.W. (2013). Corticobasal degeneration with olivopontocerebellar atrophy and TDP-43 pathology: An unusual clinicopathologic variant of CBD. Acta Neuropathol..

[127] Jacob C., Espay A.J., Hagen M.C., Duker A.P. (2016). Misleading Imaging and Clinical Features in Pathology-Proven Corticobasal Degeneration. Mov. Disord. Clin. Pract..

[128] Koga S., Sanchez-Contreras M., Josephs K.A., Uitti R.J., Graff-Radford N., van Gerpen J.A., Cheshire W.P., Wszolek Z.K., Rademakers R., Dickson D.W. (2017). Distribution and characteristics of transactive response DNA binding protein 43 kDa pathology in progressive supranuclear palsy. Mov. Disord..

[129] Flanagan E.P., Duffy J.R., Whitwell J.L., Vemuri P., Dickson D.W., Josephs K.A. (2016). Mixed tau and TDP-43 pathology in a patient with unclassifiable primary progressive aphasia. Neurocase.

[130] Tazwar M., Evia A.M., Ridwan A.R., Leurgans S.E., Bennett D.A., Schneider J.A., Arfanakis K. (2024). Limbic-predominant age-related TDP-43 encephalopathy neuropathological change (LATE-NC) is associated with abnormalities in white matter structural integrity and connectivity: An ex-vivo diffusion MRI and pathology investigation. Neurobiol. Aging.

[131] Lavrova A., Pham N.T.T., Reid R.I., Boeve B.F., Knopman D.S., Petersen R.C., Nguyen A.T., Ross Reichard R., Dickson D.W., Jack C.R. (2025). Relation of Alzheimer’s disease-related TDP-43 proteinopathy to metrics from diffusion tensor imaging (DTI) and neurite orientation dispersion and density imaging (NODDI). Neurobiol. Aging.

[132] Gatto R.G., Martin P.R., Utianski R.L., Duffy J.R., Clark H.M., Botha H., Machulda M.M., Josephs K.A., Whitwell J.L. (2024). Diffusion tensor imaging-based multi-fiber tracking reconstructions can regionally differentiate phonetic versus prosodic subtypes of progressive apraxia of speech. Cortex.

[133] Badihian N., Gatto R.G., Satoh R., Ali F., Clark H.M., Pham N.T.T., Whitwell J.L., Josephs K.A. (2024). Clinical and neuroimaging characteristics of primary lateral sclerosis with overlapping features of progressive supranuclear palsy. Eur. J. Neurol..

[134] Costa F., Gatto R.G., Pham N.T.T., Ali F., Clark H.M., Stierwalt J., Machulda M.M., Agosta F., Filippi M., Josephs K.A. (2025). Longitudinal assessment of white matter alterations in progressive supranuclear palsy variants using diffusion tractography. Park. Relat. Disord..

[135] Chouliaras L., O’Brien J.T. (2023). The use of neuroimaging techniques in the early and differential diagnosis of dementia. Mol. Psychiatry.

[136] Juengling F., Wuest F., Schirrmacher R., Abele J., Thiel A., Soucy J.P., Camicioli R., Garibotto V. (2025). PET Imaging in Dementia: Mini-Review and Canadian Perspective for Clinical Use. Can. J. Neurol. Sci..

[137] Lopez-Carbonero J.I., Garcia-Toledo I., Fernandez-Hernandez L., Bascunana P., Gil-Moreno M.J., Matias-Guiu J.A., Corrochano S. (2024). In vivo diagnosis of TDP-43 proteinopathies: In search of biomarkers of clinical use. Transl. Neurodegener..

[138] Minoshima S., Cross D., Thientunyakit T., Foster N.L., Drzezga A. (2022). (18)F-FDG PET Imaging in Neurodegenerative Dementing Disorders: Insights into Subtype Classification, Emerging Disease Categories, and Mixed Dementia with Copathologies. J. Nucl. Med..

[139] Bao W., Xie F., Zuo C., Guan Y., Huang Y.H. (2021). PET Neuroimaging of Alzheimer’s Disease: Radiotracers and Their Utility in Clinical Research. Front. Aging Neurosci..

[140] Conte M., De Feo M.S., Sidrak M.M.A., Corica F., Gorica J., Granese G.M., Filippi L., De Vincentis G., Frantellizzi V. (2023). Imaging of Tauopathies with PET Ligands: State of the Art and Future Outlook. Diagnostics.

[141] Jiao F., Wang M., Sun X., Ju Z., Lu J., Wang L., Jiang J., Zuo C. (2023). Based on Tau PET Radiomics Analysis for the Classification of Alzheimer’s Disease and Mild Cognitive Impairment. Brain Sci..

[142] Leuzy A., Chiotis K., Lemoine L., Gillberg P.G., Almkvist O., Rodriguez-Vieitez E., Nordberg A. (2019). Tau PET imaging in neurodegenerative tauopathies-still a challenge. Mol. Psychiatry.

[143] Knight A.C., Morrone C.D., Varlow C., Yu W.H., McQuade P., Vasdev N. (2023). Head-to-Head Comparison of Tau-PET Radioligands for Imaging TDP-43 in Post-Mortem ALS Brain. Mol. Imaging Biol..

[144] Carlos A.F., Tosakulwong N., Weigand S.D., Senjem M.L., Schwarz C.G., Knopman D.S., Boeve B.F., Petersen R.C., Nguyen A.T., Reichard R.R. (2023). TDP-43 pathology effect on volume and flortaucipir uptake in Alzheimer’s disease. Alzheimers Dement..

[145] Bevan-Jones W.R., Cope T.E., Jones P.S., Passamonti L., Hong Y.T., Fryer T.D., Arnold R., Allinson K.S.J., Coles J.P., Aigbirhio F.I. (2018). [(18)F]AV-1451 binding in vivo mirrors the expected distribution of TDP-43 pathology in the semantic variant of primary progressive aphasia. J. Neurol. Neurosurg. Psychiatry.

[146] Matias-Guiu J.A., Pytel V., Cabrera-Martin M.N., Galan L., Valles-Salgado M., Guerrero A., Moreno-Ramos T., Matias-Guiu J., Carreras J.L. (2016). Amyloid- and FDG-PET imaging in amyotrophic lateral sclerosis. Eur. J. Nucl. Med. Mol. Imaging.

[147] Sennfalt S., Pagani M., Fang F., Savitcheva I., Estenberg U., Ingre C. (2023). FDG-PET shows weak correlation between focal motor weakness and brain metabolic alterations in ALS. Amyotroph. Lateral Scler. Front. Degener..

[148] Caminiti S.P., De Francesco S., Tondo G., Galli A., Redolfi A., Perani D., Alzheimer’s Disease Neuroimaging I., Interceptor P. (2024). FDG-PET markers of heterogeneity and different risk of progression in amnestic MCI. Alzheimers Dement..

[149] Buciuc M., Botha H., Murray M.E., Schwarz C.G., Senjem M.L., Jones D.T., Knopman D.S., Boeve B.F., Petersen R.C., Jack C.R. (2020). Utility of FDG-PET in diagnosis of Alzheimer-related TDP-43 proteinopathy. Neurology.

[150] Tanaka M., Diano M., Battaglia S. (2023). Editorial: Insights into structural and functional organization of the brain: Evidence from neuroimaging and non-invasive brain stimulation techniques. Front. Psychiatry.

[151] Di Fazio C., Tamietto M., Stanziano M., Nigri A., Scaliti E., Palermo S. (2025). Cortico-Cortical Paired Associative Stimulation (ccPAS) in Ageing and Alzheimer’s Disease: A Quali-Quantitative Approach to Potential Therapeutic Mechanisms and Applications. Brain Sci..

[152] Palermo S., Di Fazio C., Scaliti E., Stanziano M., Nigri A., Tamietto M. (2025). Cortical excitability and the aging brain: Toward a biomarker of cognitive resilience. Front. Psychol..

[153] Alongi P., Laudicella R., Panasiti F., Stefano A., Comelli A., Giaccone P., Arnone A., Minutoli F., Quartuccio N., Cupidi C. (2022). Radiomics Analysis of Brain [(18)F]FDG PET/CT to Predict Alzheimer’s Disease in Patients with Amyloid PET Positivity: A Preliminary Report on the Application of SPM Cortical Segmentation, Pyradiomics and Machine-Learning Analysis. Diagnostics.

